# Tau Overexpression Impacts a Neuroinflammation Gene Expression Network Perturbed in Alzheimer’s Disease

**DOI:** 10.1371/journal.pone.0106050

**Published:** 2014-08-25

**Authors:** Paul D. Wes, Amy Easton, John Corradi, Donna M. Barten, Nino Devidze, Lynn B. DeCarr, Amy Truong, Aiqing He, Nestor X. Barrezueta, Craig Polson, Clotilde Bourin, Marianne E. Flynn, Stefanie Keenan, Regina Lidge, Jere Meredith, Joanne Natale, Sethu Sankaranarayanan, Greg W. Cadelina, Charlie F. Albright, Angela M. Cacace

**Affiliations:** 1 Department of Neuroscience, Bristol-Myers Squibb, Wallingford, Connecticut, United States of America; 2 Department of Applied Genomics, Bristol-Myers Squibb, Wallingford, Connecticut, United States of America; Boston University School of Medicine, United States of America

## Abstract

Filamentous inclusions of the microtubule-associated protein, tau, define a variety of neurodegenerative diseases known as tauopathies, including Alzheimer’s disease (AD). To better understand the role of tau-mediated effects on pathophysiology and global central nervous system function, we extensively characterized gene expression, pathology and behavior of the rTg4510 mouse model, which overexpresses a mutant form of human tau that causes Frontotemporal dementia and parkinsonism linked to chromosome 17 (FTDP-17). We found that the most predominantly altered gene expression pathways in rTg4510 mice were in inflammatory processes. These results closely matched the causal immune function and microglial gene-regulatory network recently identified in AD. We identified additional gene expression changes by laser microdissecting specific regions of the hippocampus, which highlighted alterations in neuronal network activity. Expression of inflammatory genes and markers of neuronal activity changed as a function of age in rTg4510 mice and coincided with behavioral deficits. Inflammatory changes were tau-dependent, as they were reversed by suppression of the tau transgene. Our results suggest that the alterations in microglial phenotypes that appear to contribute to the pathogenesis of Alzheimer’s disease may be driven by tau dysfunction, in addition to the direct effects of beta-amyloid.

## Introduction

Filamentous inclusions of the microtubule-associated protein, tau, define a variety of neurodegenerative diseases called tauopathies [1]. The autosomal dominant tauopathy, Frontotemporal dementia and parkinsonism linked to chromosome 17 (FTDP-17), results from mutations in the tau gene, demonstrating that tau is sufficient to cause neurodegeneration [2]–[5]. Alzheimer’s disease (AD) is characterized by both tau aggregates, known as neurofibrillary tangles (NFTs), and extracellular plaques composed of beta-amyloid (Aβ) [6]. Tau also appears to be required for disease progression in AD and in transgenic mutant amyloid precursor protein (APP) derived Aβ-dependent mouse models [7]–[10]. Weighted gene correlation network analysis (WGCNA) [11] of laser microdissected regions in proximity with early NFT pathology from AD patients reveals alterations in an immune function and microglial gene expression network [12]. A similar immune function network was independently identified in AD and shown to correlate with DNA variants as well as amyloid pathology [13], [14], and included genes genetically associated with AD risk [15]–[19]. These results imply that the immune gene expression network associated with AD contributes to the etiology of the disease–or at least provides a permissive environment in which the disease can progress–rather than representing a secondary response to pathology and neuronal damage [20].

A number of tau transgenic mice have been generated to study the mechanisms by which tau participates in the etiology of AD and other tauopathies [21]. The rTg4510 mouse model is particularly informative because expression of the tau transgene can be turned off, allowing for evaluation of tau-induced changes at various stages of disease progression [22]–[25]. rTg4510 mice overexpress human tau containing an FTDP-17 mutation where the proline at position 301 is mutated to a leucine (P301L). The tau (P301L) coding region is downstream of a tetracycline operon–responsive element (TRE), and expression is driven by a second transgene expressing the tetracycline-controlled transactivator (tTA) under control of the Ca^2+^/calmodulin-dependent protein kinase II α (CaMKII α) promoter. tTA constitutively induces tau expression via the TRE, but can be inactivated with doxycycline administration. Behavioral and histopathological characterization of rTg4510 mice demonstrated that overexpression of mutant human tau results in age-related increases in insoluble tau and NFTs, astrogliosis, synaptic and neuronal loss, and impairments in learning and memory [22], [24]–[31], suggesting that this model captures at least some of the key components of human disease.

In this report, we sought to develop a more comprehensive understanding of tau-mediated effects on the pathology and behavior in the rTg4510 mice by characterizing genome-wide gene expression profiles across age and brain regions in rTg4510 and control mice, including laser microdissected hippocampal subfields. In addition, we conducted behavioral studies to assess function across cognitive and non-cognitive domains. Interestingly, the predominant class of gene expression changes were the immune function networks already identified in AD. The changes were age-dependent, coinciding with decreases in markers of neuronal activity and with behavioral deficits, andcould be reversed by turning off tau expression.

## Materials and Methods

### Subjects

rTg4510 mice and littermate controls were generated by crossing TRE-htau (P301L) FVB/N (“htau”) and CaMKIIα-tTA 129S6 (“tTA”) breeders obtained from the Mayo Clinic (Jacksonville, FL). rTg4510 mice were compared to littermate control subjects containing only the tTA transgene, only the htau transgene, or neither transgene (Double Negative; “DN”). Female mice were assessed for age-dependent changes in phenotype starting at 1.9 months of age. Animals were group-housed in colony rooms maintained at constant temperature (21±2°C) and humidity (50±10%). The rooms were illuminated 12 hours per day (lights on at 06h00). Animals had *ad libitum* access to food and water throughout the studies. Behavioral studies were conducted between 08h00 and 15h00. Doxycycline was administered via drinking water for 2 days along with 200 ppm doxycycline in their chow, as previously described [23]. Animals were maintained in accordance with the guidelines of the Animal Care and Use Committee of Bristol-Myers Squibb Company, the “Guide for Care and Use of Laboratory Animals” and the guidelines published in the National Institutes of Health Guide for the Care and Use of Laboratory Animals. Research protocols were approved by the Bristol-Myers Squibb Company Animal Care and Use Committee.

### Laser capture microdissection

Mouse brains were quickly removed and frozen on a bed of powdered dry ice. The brains were sectioned at a thickness of 10 µm, mounted onto pre-chilled polyethylene naphthalate (PEN) membrane slides (Leica Microsystems, Buffalo Grove, IL), and stored at −80°C until further processing. The staining of tissue was performed using an LCM Staining Kit (Ambion, Grand Island, NY) with some modifications to the manufacturer’s instructions. A slide chamber containing 95% ethanol was used to transport a tissue slide from the freezer to the workbench. The slide was then transferred through a series of decreasing ethanol concentrations (95%, 75%, 50%), agitating for 30–40 seconds at each concentration and blotting in between. The slide was laid flat and the tissue was covered with 200 µl of supplied cresyl violet solution for 30 seconds. The slide was then tilted and drained of the staining solution, and immersed through a series of increasing ethanol concentrations (50%, 75%, 95%, 100% twice) for 30–40 seconds each without agitation. The slide was carefully blotted dry, taking care not to disturb the PEN membrane and tissue section, and placed in a slide box containing desiccant sealed with Parafilm for 15 minutes. Processed slides were then laser microdissected using the LMD6000 instrument (Leica Microsystems, Buffalo Grove, IL). Hippocampal subfields were dissected and the tissue was collected in RLT Buffer from the RNeasy Plus Micro Kit (Qiagen, Valencia, CA).

### Gene expression profiling

RNA was isolated from frozen cortices and hippocampi using RNeasy Mini Kits (Qiagen, Valencia, CA), according to the manufacturer’s protocol. The integrity of the RNA was determined using the Agilent Bioanalyzer (Agilent Technologies, Santa Clara, CA). The RNA integrity numbers (RIN) ranged from 8.90 to 10 out of a scale of 10. All total RNA were amplified and labeled with Ovation Pico WTA System and Encore Biotin Module (Nugen INC, San Carlos, CA, USA). Labeled cDNA was hybridized on the Affymetrix Genechip HT-MG-430A (Affymetrix, Santa Clara, CA). All array hybridization, washing and scanning was performed according to the manufacturer’s recommendations.

Gene expression values were normalized with the anti-log RMA Irizarry algorithm. Statistical analysis was performed using Partek Genomics Suite 6.5 (Partek Incorporated, St. Louis, MO). To determine genes with differential expression amongst groups, a 1-way ANOVA model using Methods of Moments [32] was applied: Y_ij_ = µ + Group_i_ + ε_ij_, where Y_ij_ represents the j^th^ observation on the i^th^ Group, µ is the common effect for the whole experiment, and ε_ij_ represents the random effect present in the j^th^ observation on the i^th^ Group. The errors ε_ij_ were assumed to be normally and independently distributed with mean 0 and standard deviation δ for all measurements. To determine differences between two groups, the Fisher’s Least Significant Difference (LSD) method [33] was employed. Genes were considered differentially expressed if they met a false discovery rate cutoff of 0.05 using the step up method [34], with no fold-change cutoff.

In order to understand the biological significance of differentially expressed genes, genes from the data sets that met a False Discovery Rate cutoff of 0.05 were subjected to pathway analysis using Ingenuity Pathway Analysis (IPA, Ingenuity Systems, Redwood City, CA). Pathway analysis identified the pathways from the IPA library of canonical pathways, disease processes, and biological processes, as well as customized pathways, that were most significant to the data set. The significance of the association between the data set and the pathways was measured using the Fisher’s exact test.

Heat maps were generated by shifting the gene expression intensities to a mean of zero and scaling to a standard deviation of one. Hierarchical clustering was conducted using Partek Genomics Suite 6.5 (Partek Incorporated, St. Louis, MO), while other heat maps were generated using the Conditional Formatting function in Microsoft Excel (Microsoft, Redmond, WA).

### Accession codes

Gene expression data have been deposited in the Gene Expression Ominibus (GEO; http://www.ncbi.nlm.nih.gov/geo/) database under the accession numbers GSE56772, GSE57528 and GSE57583.

### qRT-PCR

cDNA was generated by reverse transcribing 300 ng of cortical or hippocampal RNA using the High Capacity RNA-to-cDNA Kit (Applied Biosystems, Carlsbad, CA) according to the manufacturer’s protocol. qPCR was conducted in triplicate for each target gene and each sample on a 7900 Fast Real-Time PCR System (Applied Biosystems, Carlsbad, CA) using the following cycling parameters: 1x (95°C 10 minutes), 40x (95°C 25 seconds+60°C 1 minute). The target genes were amplified using Taqman Gene Expression Assays (Applied Biosystems, Carlsbad, CA). Gene expression levels were determined using the ΔΔCt relative quantification method. Specifically, the technical replicates of the cycle threshold (Ct) values were averaged for each sample and the average Ct for the reference gene (β-actin) was subtracted from the average Ct of the target gene to give the ΔCt value for each sample. Coefficients of variation were much smaller for technical replicates (0.00089–1.56%) than for biological replicates (6.05–23.86%), allowing for the use of technical replicate averages for statistical analysis. To calculate the ΔΔCt, the average ΔCt of the vehicle-treated animals was subtracted from the average ΔCt of the experimental sample. To calculate relative expression, the formula 2^−ΔΔCt^ was used, which assumes doubling of the amplicon every amplification cycle.

### ELISA and protein quantitation

Doxycycline-treated and untreated rTg4510 and tTA mouse brains were homogenized in ice-cold RIPA buffer containing 1x protease inhibitor cocktail (Roche Diagnostics, Indianapolis, IN), 1x phosphatase inhibitor sets I and II (Calbiochem/EMD Millipore, Billerica, MA) at 10 ml/g with a Polytron homogenizer. The homogenates were incubated on ice for 15 minutes, then spun at 14,000×g for 15 minutes at 4°C. The protein level of the supernatants was verified using the BCA Protein Assay Reagent (Pierce/Thermo Scientific, Rockford, IL). The supernatants were tested in a Glial Fibrillary Acid Protein ELISA (Biovendor, Candler, NC) at 0.08 µg protein/100 µl supplied dilution buffer/well for the rTg4510 positive animals and 0.25 µg protein/100 µl supplied dilution buffer/well for the tTA alone and DN animals. The supernatants were also tested in an SPP-1 (osteopontin) ELISA (IBL, Minneapolis, MN) at 15 µg protein/100 µl supplied dilution buffer/well. The samples were tested in duplicate and the results were reported as ng GFAP or osteopontin, as read off of a standard curve, per mg of total protein loaded on the ELISA plate.

80 µl of mouse plasma from each treated animal was shipped on dry ice to Rules Based Medicine (Austin, TX) for analysis in their inflammatory antigen panel (RodentMAP 2.0– Antigens). Of the 58 analytes analyzed, data was returned for 36 assays with values above the lower limit of detection of the assay. The following analytes were detected: eotaxin, fibrinogen, GCP2, haptoglobin, IgGA, IL-7, mCSF1, MDC, MCP1, myoglobin, SAP, VEGFA, Apo A1, CD40, CD40L, CRP, factor VII, basic FGF, IP-10, IL-18, LIF, lymphotactin, MIP-1a, MIP-1b, MIP-1g, MIP-3b, MMP-9, MCP-3, MCP-5, MPO, SCF, TPO, TF, TIMP-1, VCAM-1, VWF.

### Immunohistochemistry

Brain tissue was drop-fixed in 4% paraformaldehyde (PFA) and processed through 15% and 30% sucrose. 40 µm sections were collected using a sliding microtome and stored in cryoprotectant solution. Sections were rinsed in phosphate-buffered saline (PBS) to remove cryoprotectant. Sections were post-fixed in 3.7% formaldehyde in PBS for 10 minutes and washed using PBS (or washed in PBS alone). Endogenous peroxidase activity was removed by incubation in 3% hydrogen peroxide/10% methanol in PBS for 30 minutes. After washing in PBS, slides were blocked in 5–10% normal goat serum/0.1–0.3% triton X-100 in PBS for 60 minutes. Sections were then incubated with antibodies against human tau (HT7-biotin, 1∶3,000, Thermo Scientific, Rockford, MD), Iba-1 (1∶60,000, Wako Chemicals USA, Richmond, VA), glial fibrillary acidic protein (GFAP, 1∶20,000, Dako, Carpinteria, CA), CD68 (1∶1000) or CD11b (1∶2000) (Abcam Inc. Cambridge, MA), diluted in the blocking solution, overnight at 4°C. Following washing in PBS, sections were also incubated with biotinylated anti-rabbit IgG antibodies (Vector Labs, Burlingame, CA) for 1 hour at room temperature. Sections were developed using a Vectastain ABC Elite Kit (with or without 0.1% triton X-100, Vector Labs, Burlingame, CA) for 1 hour followed by detection with diaminobenzidine reagent with or without nickel intensification (Vector Labs, Burlingame, CA).

### Behavioral testing

#### Spontaneous locomotor activity

Following acclimation to the testing room, subjects were placed in the testing apparatus for 60 minutes for automated locomotor activity recording. The testing chamber was a Plexiglas box (25×25×41 cm) equipped with a TruScan activity monitor (Coulbourn Instruments, Allentown, PA) which detects interruptions of 16 photobeams spaced 1.5 cm apart and 0.8 cm above the floor. Locomotor activity was assessed under low lighting conditions (100 lux). Activity was collected in 250 msec intervals and summed for the total 60 minute period. The data were expressed as total distance traveled (in cm).

#### Short term spatial working memory

Subjects were placed into the stem of a Y-shaped maze and allowed to explore the maze for 8 minutes. The maze was made of gray polyvinyl choride (PVC) and each arm was 40 cm in length with walls 24 cm high. The order of arm entry and total number of arm entries was recorded manually. An entry was scored when all four limbs of the animal entered a given arm. A sequence of entering 3 different arms (e.g. 3,2,1 or 2,1,3) was considered one alternation. The total number of alternations was recorded during the 8 minute testing session and expressed as a percentage of total arm entries made. The percent alternation is generally considered an index of spatial working memory and can be disrupted with lesions to frontal cortex, hippocampus, and septal brain regions [35], [36]. The total number of arm entries was recorded as a measure of locomotor activity. Highly repetitive search patterns in the Y maze were described using a stereotypy index, which was calculated from the number of alternations which were repeating in sequence (e.g. 3,2,1,3,2,1,3,2,1 = score of 3) throughout the test session.

#### Novel object recognition

The object recognition task is based on the spontaneous behavior of rodents to explore a novel object more than a familiar one [37], [38]. After a one hour retention interval between training (T1) and testing (T2) sessions, mice remembering the objects from the training session will show a preference for the novel object on the test session. For these experiments, animals were handled for 3 days and habituated to the chamber (48 cm×38 cm×20 cm) on the day prior to the test session. The chamber was made of polyethylene and lined with vinyl flooring. On the test day, animals were placed in the rectangular test chamber and allowed to explore two identical objects (7.6 cm high×5.1 cm wide) for a 15 minute training period. One hour later, mice were placed back into the test chamber for a 10 minute test session, this time with one object they had observed during training and one novel object. Objects were cleaned thoroughly with 25% ethanol between training and testing sessions and between subjects, and were cleaned again at the end of the day with mild detergent. Object exploration was only considered when the animal’s nose was pointed at the object. Exploration was recorded using ObjectScan tracking software (Cleversys, Reston, VA). Data are reported as percent of time spent exploring objects (i.e. novel time/novel+familiar time*100 or familiar time/novel+familiar time*100) and as a discrimation ratio (i.e. novel-familar time/novel+famliar time).

#### Behavioral statistics

Statistical analyses for all behavioral tests were conducted in GraphPad Prism (GraphPad Software, Inc., La Jolla, CA) using an ANOVA followed by a Tukey’s Multiple Comparisons Test when appropriate. For novel object recognition, data were also analyzed using either a 2-way ANOVA followed by Bonferroni’s post-hoc tests on percent exploration time or a 1-way ANOVA followed by Tukey’s post-hoc tests on the discrimination ratio.

## Results

### 

#### Gene expression profiling

**Figure 1:**
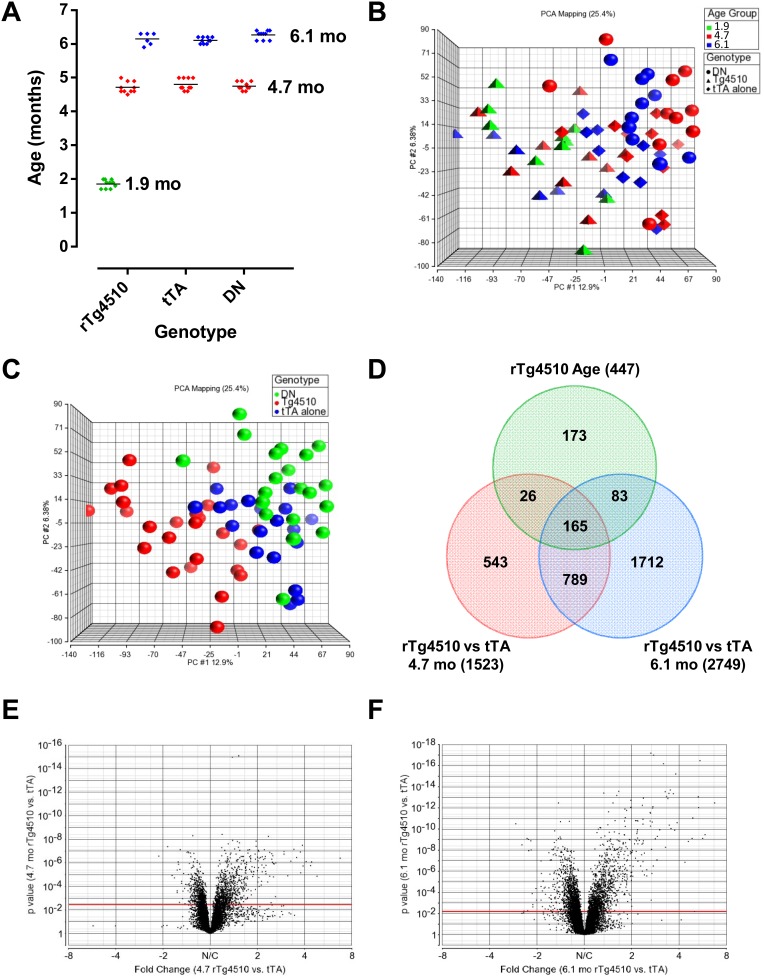
Identification of age-dependent gene expression changes in rTg4510 females. (A) Animals available for analysis included age groups averaging 1.9 months, 4.7 months and 6.1 months, respectively. The 1.9 month old age group was only available for the rTg4510 genotype, while the other age groups were available for all genotypes. (B) Principle Component Analysis (PCA) of full genome expression data of all samples was used to identify any outliers in the sample set. One outlier (6.1 month tTA, not shown) was removed for all analyses. Samples are colored by age (green: 1.9 months; red: 4.7 months; blue: 6.1 months). Genotypes are represented by shapes (spheres: DN; pyramids: rTg4510; octahedra: tTA). (C) Principle Component Analysis (PCA) with samples colored by genotype (green: DN; blue: rTg4510; red: tTA) highlight differences amongst all three genotypes. (D) 447 probe sets were altered as a function of age in rTg4510 females (“rTg4510 Age”, 1-way ANOVA of 1.9, 4.7 and 6.1 month old rTg4510 females, FDR<0.05, no fold-change cutoff). 1523 probe sets were different between rTg4510 and tTA animals at 4.7 months (“rTg4510 vs tTA 4.7 mo”, 1-way ANOVA of 4.7 month old DN, tTA and rTg4510 females followed by Fisher’s Least Significant Difference [LSD] test comparing rTg4510 and tTA, FDR<0.05, no fold-change cutoff), while 2749 probe sets were different at 6.1 months (“rTg4510 vs tTA 6.1 mo”, 1-way ANOVA of 6.1 month-old DN, tTA and rTg4510 females followed by Fisher’s Least Significant Difference [LSD] test comparing rTg4510 and tTA, FDR<0.05, no fold-change cutoff). The intersection of these 3 datasets contained 165 probe sets, representing 139 genes, which define the genes that are significantly altered with age in rTg4510 animals compared to control tTA animals. A 2-way ANOVA to look at an interaction between age and genotype could not be used for this dataset because data for all age groups were not available for all genotypes. (E) Volcano plot of p-value versus fold change of all genes comparing 4.7 month old rTg4510 and tTA animals. The FDR<0.05 cutoff is indicated with a red line. (F) Volcano plot of p-value versus fold change of all genes comparing 6.1 month old rTg4510 and tTA animals. The FDR<0.05 cut-off is indicated with a red line.

rTg4510 mice display progressive increases in tau pathology, sarkosyl insoluble total tau, and sarkosyl insoluble hyperphosphorylated tau from 2 to 6 months of age [22]–[25], [31]. To begin to characterize the effects of mutant tau overexpression on brain function, we analyzed gene expression patterns in the hippocampi of female rTg4510, tTA and DN (Double Negative; littermates containing neither transgene) mice using full genome microarrays (Figure 1A). Principle component analysis (PCA) was used to identify the major sources of variation in the dataset, and to identify any outliers (Figures 1B–C). One outlier was identified and excluded from all analysis (not shown). Both age and genotype contributed to variation in the dataset, including large differences between the tTA and DN controls. tTA littermates were therefore chosen as the key control, since this genotype differs from rTg4510 only by the presence of the tau transgene. 1,523 and 2,749 probe sets showed altered expression between rTg4510 and tTA mice at 4.7 months and 6.1 months, respectively (Fisher’s Least Significant Difference test between rTg4510 and tTA mice at each age, False Discovery Rate, FDR<0.05); 447 probe sets showered altered expression in rTg4510 animals as they aged from 1.9 to 6.1 months (rTg4510 ANOVA of all three age groups, False Discovery Rate, FDR<0.05). Of the probe sets with altered expression between rTg4510 and tTA mice, 165 probe sets, representing 139 genes, also showed age-dependent changes in rTg4510 animals (Figure 1D, Table S1 in File S1). No fold-change cutoff was selected, because defining the genes of interest by using an intersection between three comparisons reduced the chances of introducing false positives. The distribution of fold-changes for the 4.7 month and 6.1 month old rTg4510 females compared to tTA controls is shown in Figures 1E and 1F, respectively, showing a correlation with the magnitude of the expression difference with the significance of the p value, as expected.

**Figure 2:**
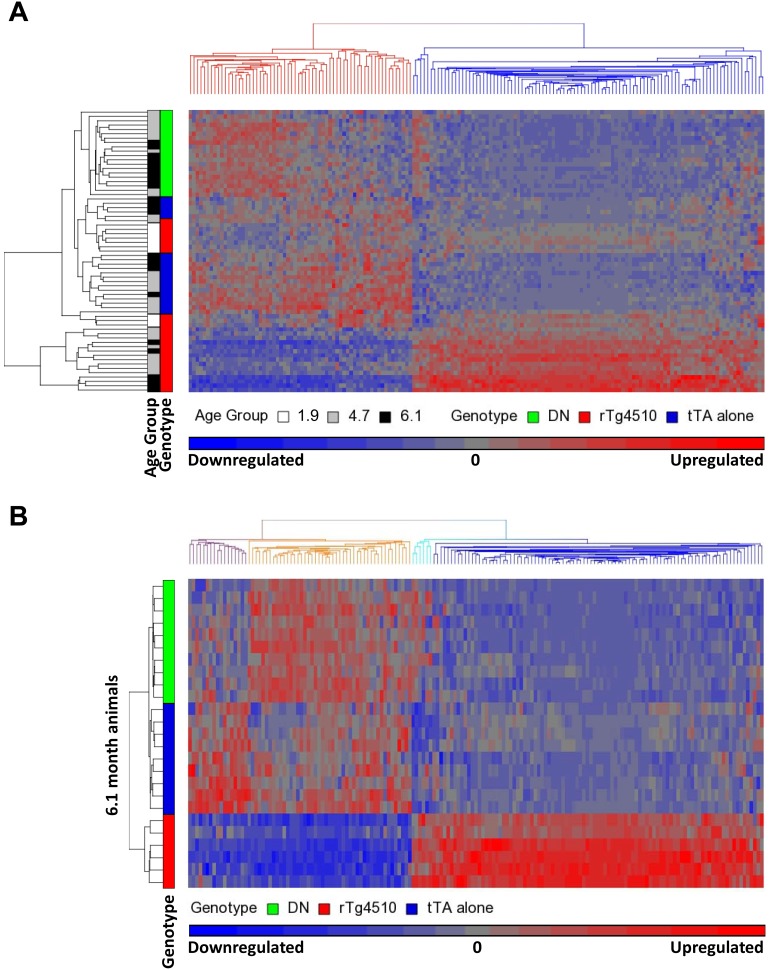
Heirarchical clustering of age-dependent gene expression changes in rTg4510 mice. Heirarchical clustering of the 165 probe sets that showed age-dependent changes in rTg4510 mice (A) using all ages and genotyopes analysed, or (B) only 6.1 month old animals, illustrates probe sets that were either downregulated or upregulated with age in rTg4510 animals. Standardization was achieved by shifting the expression values to a mean of zero and scaling to a standard deviation of one.

Hierarchical clustering was performed in order to understand the pattern of the gene expression changes across groups. The 165 probe sets clustered primarily into modules that were either downregulated or upregulated with age in rTg4510 mice compared to both DN and tTA genotypes (Figure 2A–B).

**Figure 3:**
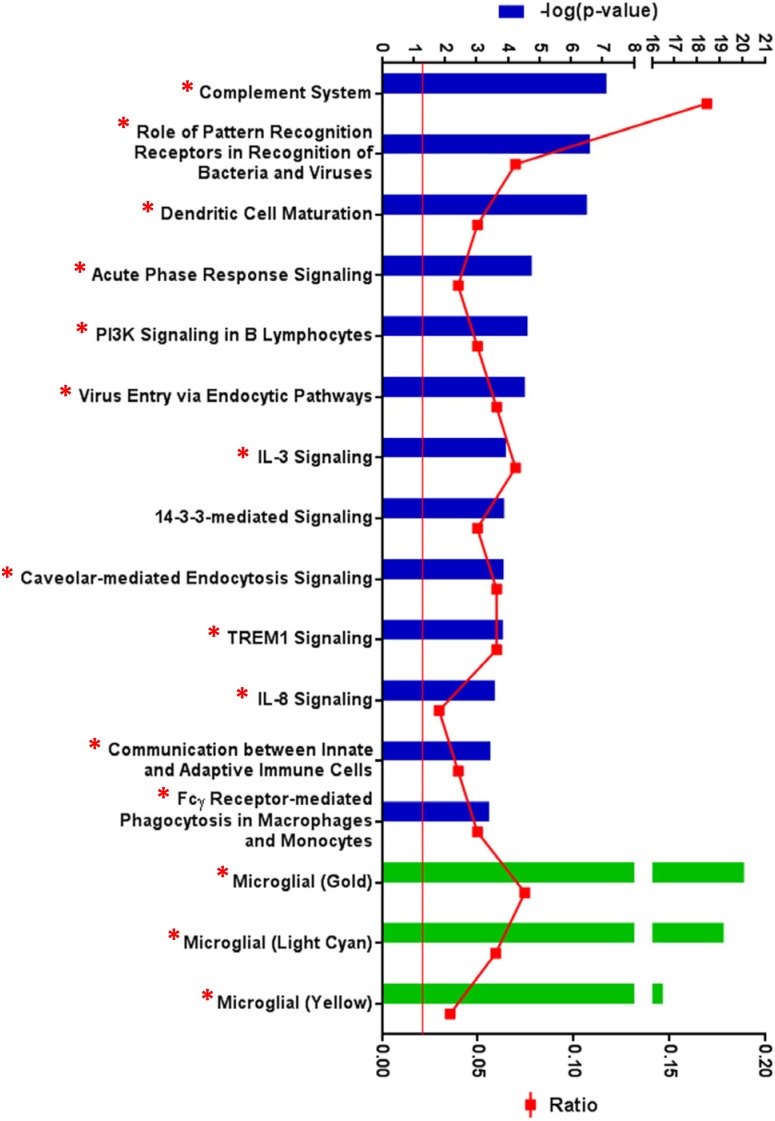
Inflammatory pathways are overrepresented by age-dependent gene expression changes in rTg4510 animals. The 139 genes whose expression change with age in rTg4510 animals were subjected to Ingenuity Pathway Analysis. The top axis represents the –log(p-value) of the probability that the gene set represented the canonical pathway by chance, as determined by the Fisher’s Exact Test, with the red line delineating p = 0.05 threshold, and the bottom axis represents the ratio of the number of genes from the query gene set in the pathway to the total number of genes in the pathway. The top canonical pathways, shown by the blue bars, significantly overrepresented by these genes fell predominantly in pathways involved in inflammatory responses, as indicated by the red asterices. The green bars show that these genes also significantly overlap with gene expression modules affected in AD [14]. Gene expression modules identified by weighted gene correlation network analysis (WGCNA) are named by assigning arbitrary colors [11], in this case gold, light cyan and yellow. The genes that fell into these pathways are shown in Table S3 in File S1.

**Figure 4:**
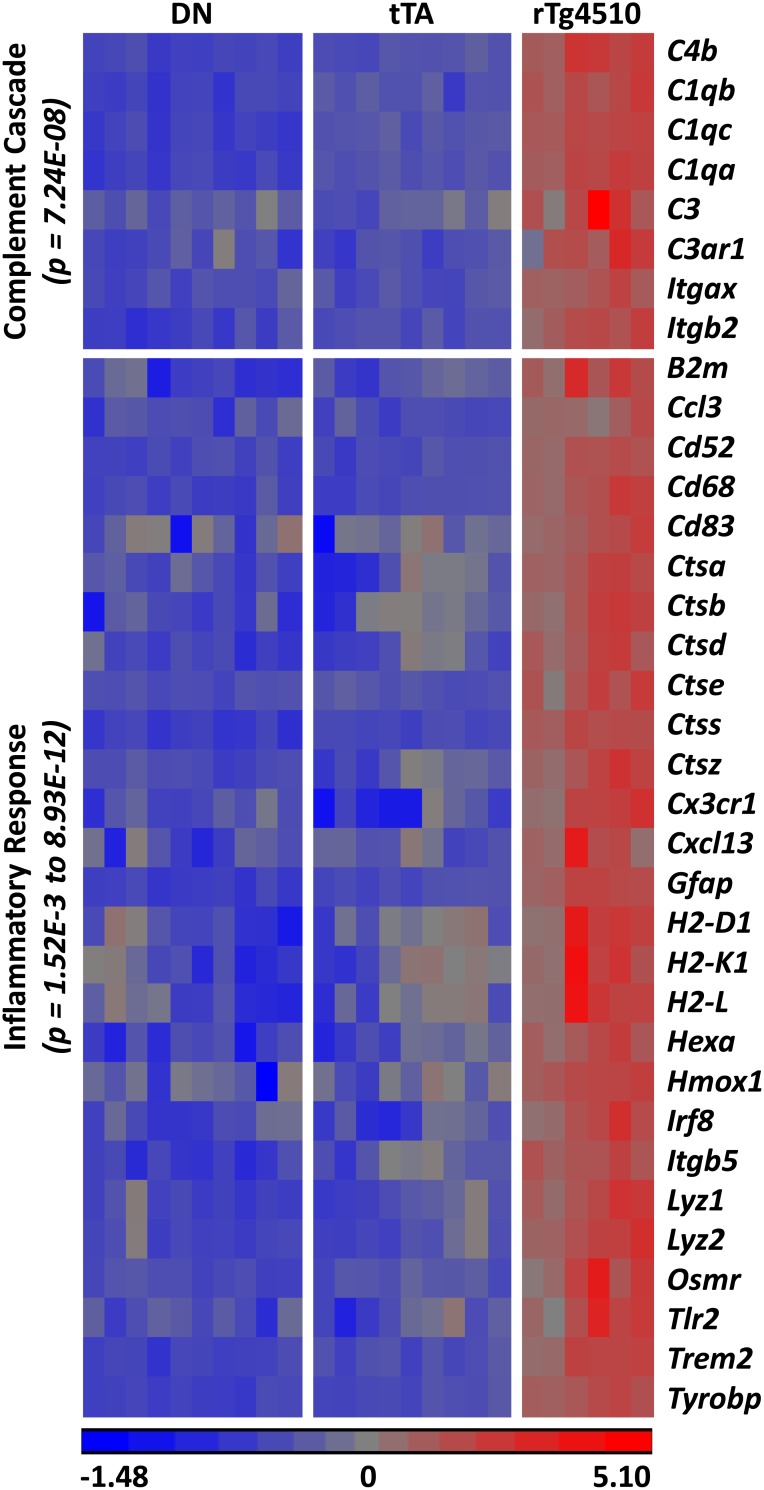
Key inflammatory gene expression changes in 6.1 month old rTg4510 females. Heat map of key inflammatory genes with age-dependent expression changes in rTg4510. Data for only 6.1 month old animals are shown. Pathway analysis revealed that the complement cascade was the most significantly affected canonical pathway in the IPA library (p = 7.24×10^−8^). However, IPA does not include *Itgax* (CD11c) and *Itgb2* (CD18)–which encode subunits of complement receptors–in the canonical complement pathway, so the p value for the complement cascade is underestimated. Many of the other genes fell into various inflammatory response pathways, with p values ranging from 1.52×10^−3^ to 8.93×10^−12^. Standardization was achieved by shifting the expression values to a mean of zero and scaling to a standard deviation of one.

**Figure 5:**
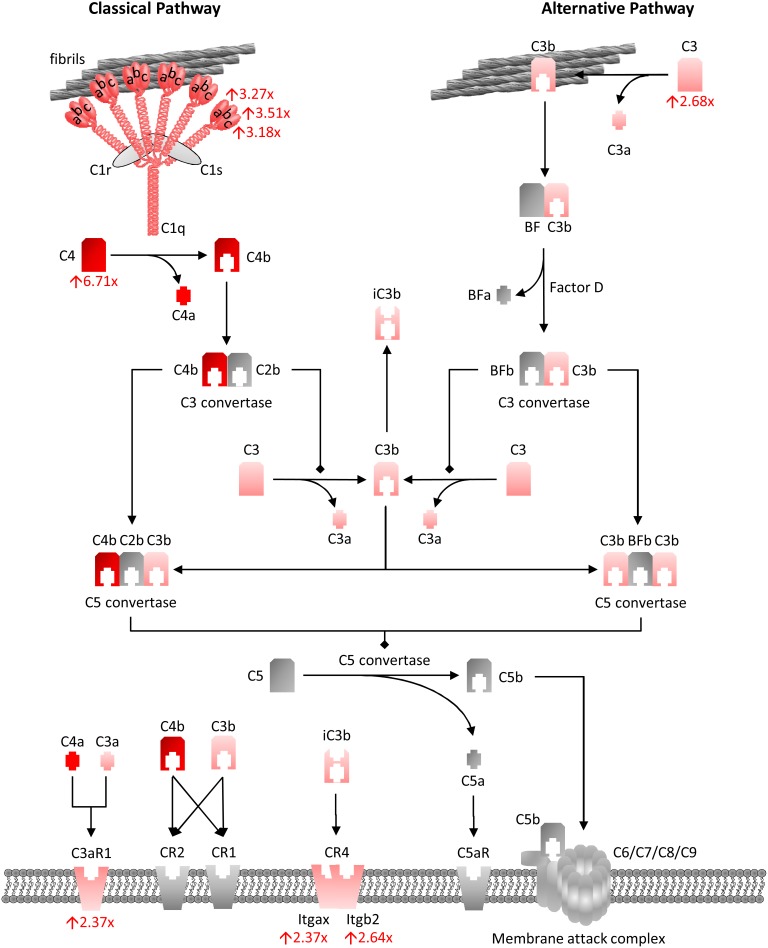
Upregulation of the complement system in rTg4510 mice. The complement cascade, shown here adapted from the IPA library, is the most significant pathway overrepresented by genes with age-dependent changes in expression in rTg4510 hippocampus. All gene expression changes in this pathway were increases in expression. The degree of shading in red corresponds to the amount of upregulation in rTg4510 hippocampus compared to tTA at 6.1 months of age, with the numbers indicating the fold-change.

The biological roles of the 139 genes with altered age-dependent expression in rTg4510 females were investigated by subjecting them to Ingenuity Pathway Analysis. The most significant disease and biological functions of this set of genes was inflammatory responses, with p values ranging from 1.52×10^−3^ to 8.93×10^−12^ (Table S2 in File S1). Similarly, the top canonical pathways overrepresented by these genes were predominantly inflammatory pathways (Figure 3, Table S3 in File S1). Some of the key inflammatory genes driving these associations are shown in Figure 4. The complement pathway was the most significantly impacted canonical pathway in rTg4510 mice (Figure 4–5).

**Figure 6:**
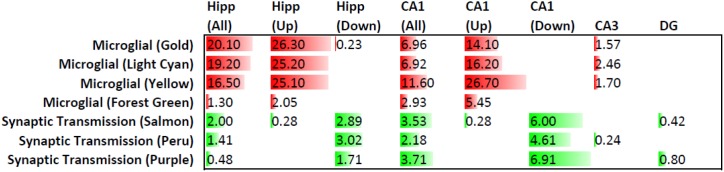
Relationship of gene expression changes in rTg4510 compared to AD brains. Genes with differential expression in rTg4510 brains were compared to gene expression modules affected in AD [14] using the Fisher’s Exact Test. Query samples included all the 139 differentially expressed genes in hippocampus, “Hipp (All)”, the 82 that were upregulated, “Hipp (Up)”, the 57 that were downregulated, “Hipp (Down)”, as well as the genes that were impacted in the CA1, DG, and CA3 microdissected samples. Numbers represent the probability, expressed as –log(p) values, that the query gene sets overlapped with the AD gene expression modules by chance, with –log(p) >1.30 (i.e. p<0.05) serving as the cutoff for significance. When no numbers are indicated, no genes in the query set were present in the modules. The microglial modules (shown in red) were most affected in the Hipp (All), Hipp (Up) and CA1(Up) datasets, whereas the Synpatic Transmission modules (shown in green) were mode affected in the Hipp (Down), CA1(All) and CA1(Down) datasets. Gene expression modules identified by weighted gene correlation network analysis (WGCNA) are named by assigning arbitrary colors [11], in this case gold, light cyan, yellow, forest green, salmon, peru and purple.

**Figure 7:**
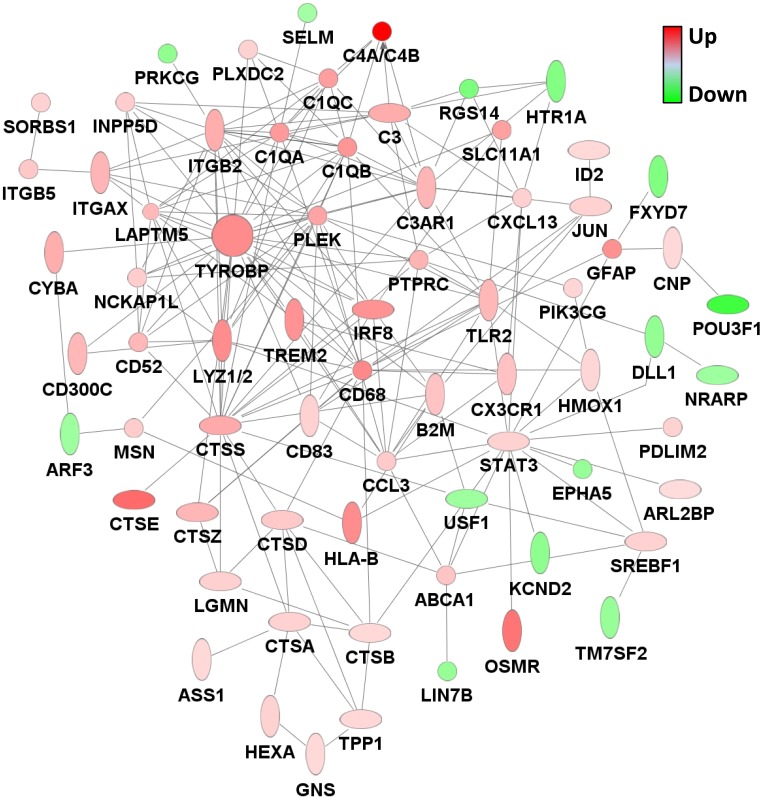
Microglial gene expression network altered in rTg4510 mice. Most of the genes with age-dependent gene expression changes in rTg4510 mice compared to controls fall into an interconnected network of genes related to microglial function. TYROBP forms a major hub of this network. Colors indicate levels of gene expression changes in rTg4510 mice at 6.1 months of age compared to tTA controls, with red indicating upregulation and green indicating downregulation. The network was generated in Ingenuity Pathway Analysis and additional interactions were added using STRING (Search Tool for the Retrieval of Interacting Genes/Proteins) [63].

One of the top genes to change expression with age in rTg4510 mice, *Trem2*, is also genetically associated with AD [16], [17]. To further relate gene expression changes in rTg4510 animals to human neurodegenerative disease, we used the Fisher’s Exact Test to compare the 139 affected genes to gene expression modules that were identified in AD using Weighted Gene Correlation Network Analysis (WGCNA) [14]. Interestingly, the 139 genes showed significant overlap with several of the microglial gene expression modules identified in AD, with p values ranging from 3.16×10^−17^ and 7.94×10^−21^ (Figure 3, 6, Table S3 in File S1). Furthermore, this gene set showed significant overlap with AD-related synaptic transmission modules, albeit with much higher p-values (p = 0.010 and 0.039, Figure 6, Table S3 in File S1). Almost all the genes overlapping with the microglial modules were upregulated with age in rTg4510, and almost all the genes overlapping with the synaptic transmission modules were downregulated (see Table S1 in File S1). When the upregulated and downregulated genes were compared with the AD gene expression modules separately, the significance of the overlap with the respective modules improved substantially, particularly for the microglial modules (Figure 6). The identities of the genes that fall into the different modules are shown in Table S1 in File S1. Ingenuity network analysis of all the genes with altered age-dependent expression in rTg4510 hippocampus resulted in a network very similar to the gene expression networks observed in AD, with TYROBP and CD68 forming major hubs (Figure 7) [14].

**Figure 8:**
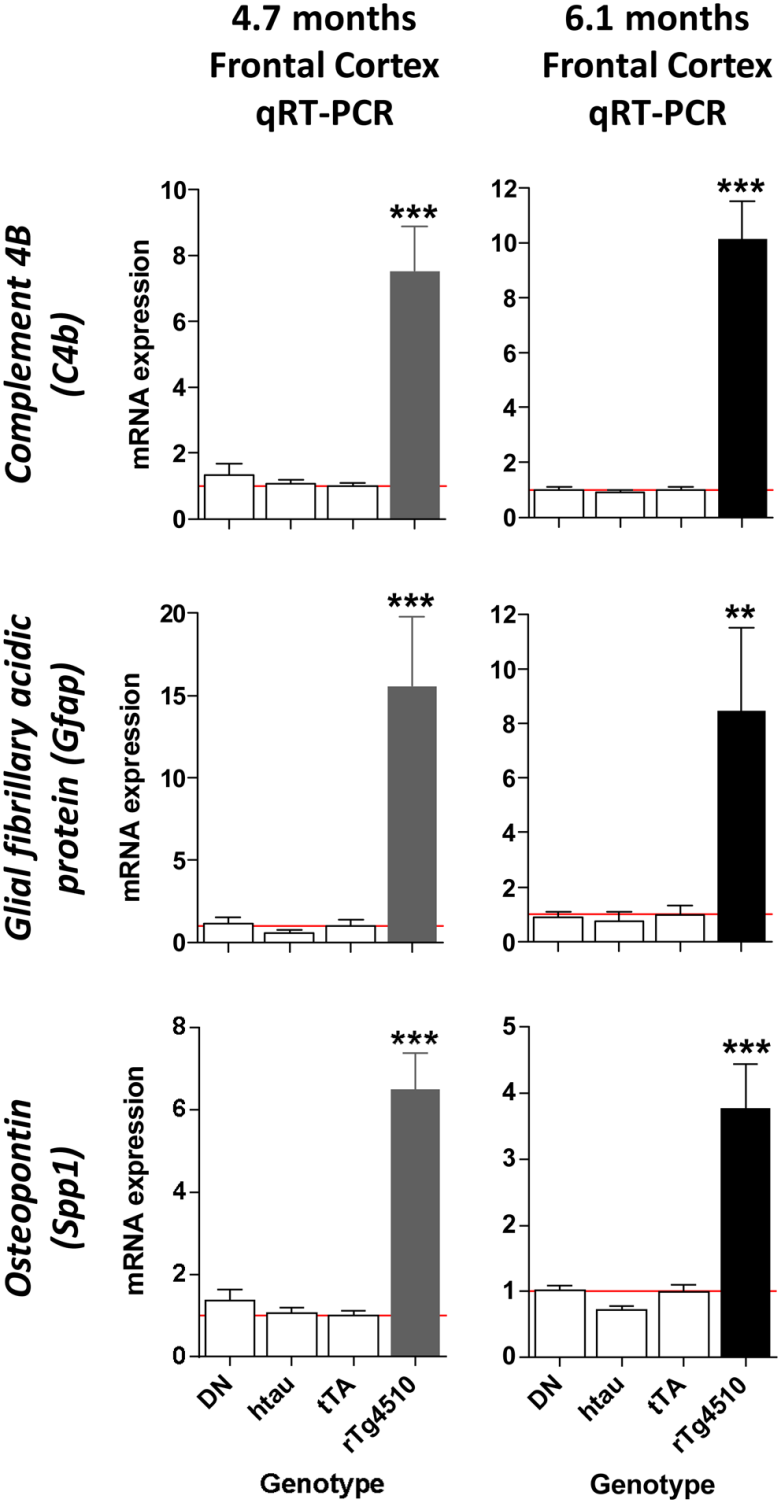
Inflammatory genes upregulated in the rTg4510 frontal cortex. *C4b*, *Gfap* and *Spp1* mRNA expression levels were higher in rTg4510 animals compared to all other genotypes in the frontal cortex, as determined by qRT-PCR. mRNA expression levels are all normalized to tTA. **p<0.01, ***p<0.001 compared to tTA using the Dunnett multiple comparison test. Error bars indicate SEM.

**Figure 9:**
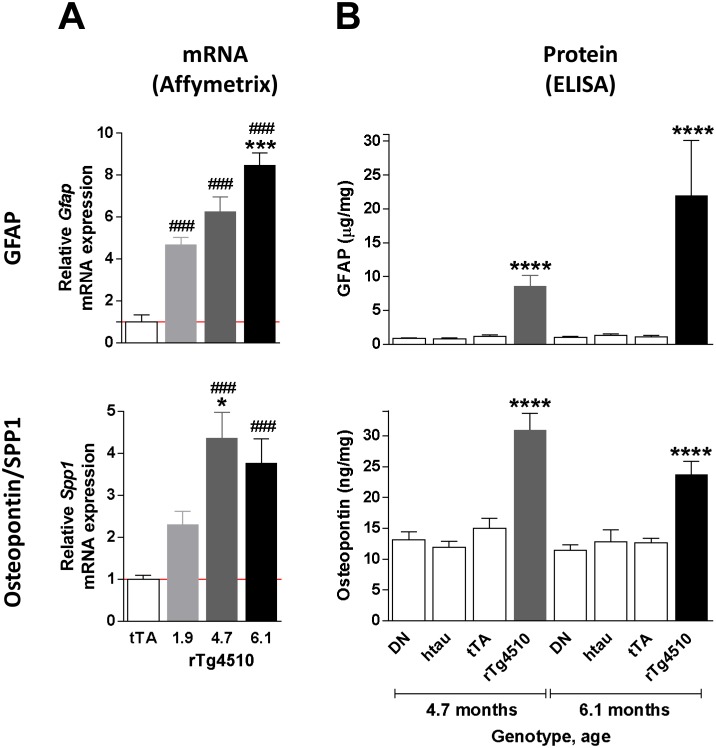
Upregulation of neuroinflammation markers in the rTg4510 brain. (A) *Gfap* and *Spp1* (which encodes for osteopontin) were upregulated at the mRNA level, as measured on Affymetrix microarrays, in rTg4510 hippocampus. *Gfap* expression increased progressively across the 3 age groups, whereas *Spp1* increased between 1.9 and 4.7 months of age, but did not differe significantly between 4.7 and 6.1 months of age. Expression levels are normalized to 6.1 month old tTA animals. Significance was determined using a one-way ANOVA followed by the Dunnett multiple comparison test. *p<0.05, ***p<0.001 compared to 1.9 month-old rTg4510. ^###^p<0.001 compared to tTA. (B) GFAP and osteopontin protein levels, as measured by ELISA, were significantly highers in the cortex of rTg4510 animals at 4.6 and 6.1 months. Significance was determined using a one-way ANOVA followed by the Dunnett multiple comparison test. ****p<0.0001 compared to tTA of the respective ages. Error bars indicate SEM.

**Figure 10:**
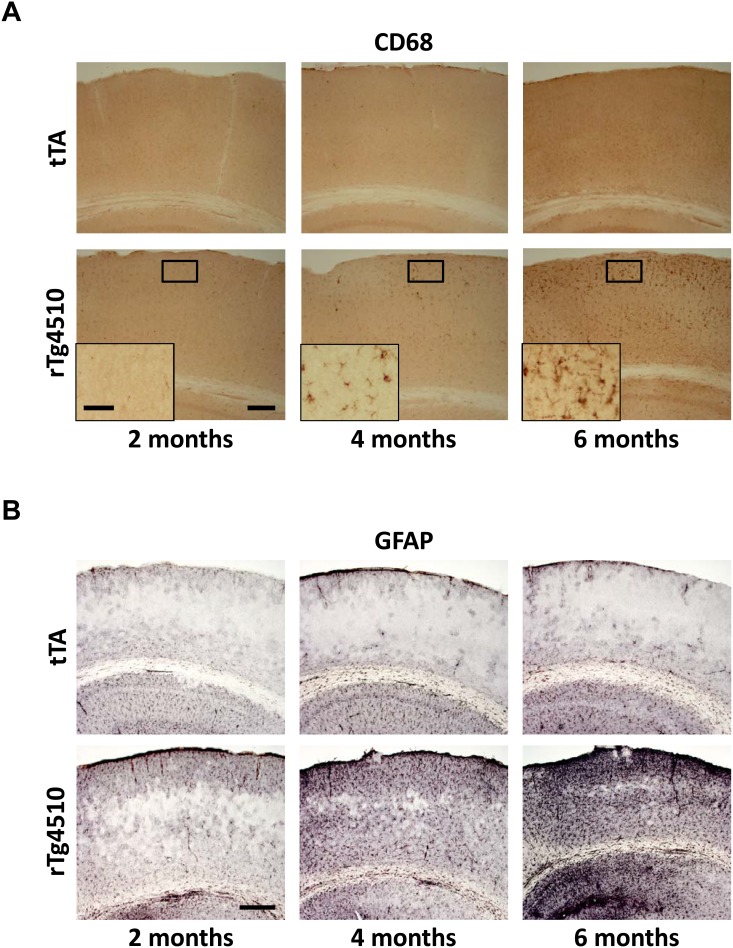
Immunohistochemistry of microglial and astrocytic markers. Cortical staining for (A) the microglial marker, CD68, and (B) the astrocytic marker, GFAP, increased as a function of age in rTg4510 animals, but remained unchanged in tTA animals. Scale bar, 200 µm, inset scale bar, 50 µm.

To confirm the gene expression profiling results and determine the extent of neuroinflammation in rTg4510 mice, expression levels of key neuroinflammatory genes and their protein products were evaluated by qRT-PCR, ELISA and immunohistochemistry in the cortex. Expression of *complement 4B (C4b), glial fibrillary acidic protein (Gfap)* and *osteopontin (Spp1)*, which represent neuroinflammatory changes in various cell types, including microglia, astrocytes, and possibly infiltrating monocytes and other immune cells, were also found to be dramatically upregulated in the frontal cortex by qRT-PCR (Figure 8). Upregulation of GFAP and osteopontontin in the cortex was further confirmed at the protein level by ELISA (Figure 9). To examine the morphological and anatomical changes in myeloid cells (which may include microglia or infiltrating monocytes) and astrocytes, brain sections were stained for CD68 and GFAP, respectively (Figure 10). These antigens were chosen because they are well-characterized antigens forimmunohistochemistry, and were upregulated at the gene expression level in rTg4510 animals. Both CD68 and GFAP showed increases in staining with age in rTg4510 mice, but did not change in control tTA mice. CD11b, a microglial marker that showed modest upregulation at the gene expression level, also showed a modest increase in signal in rTg4510 brain with age by immunohistochemistry (Figure S1).

We chose to focus our gene expression studies on female mice to avoid any confounds caused by gender differences. Future studies will be required to determine whether gene expression changes in rTg4510 animals differ between males and females. *Suppression of tau expression.*

**Figure 11:**
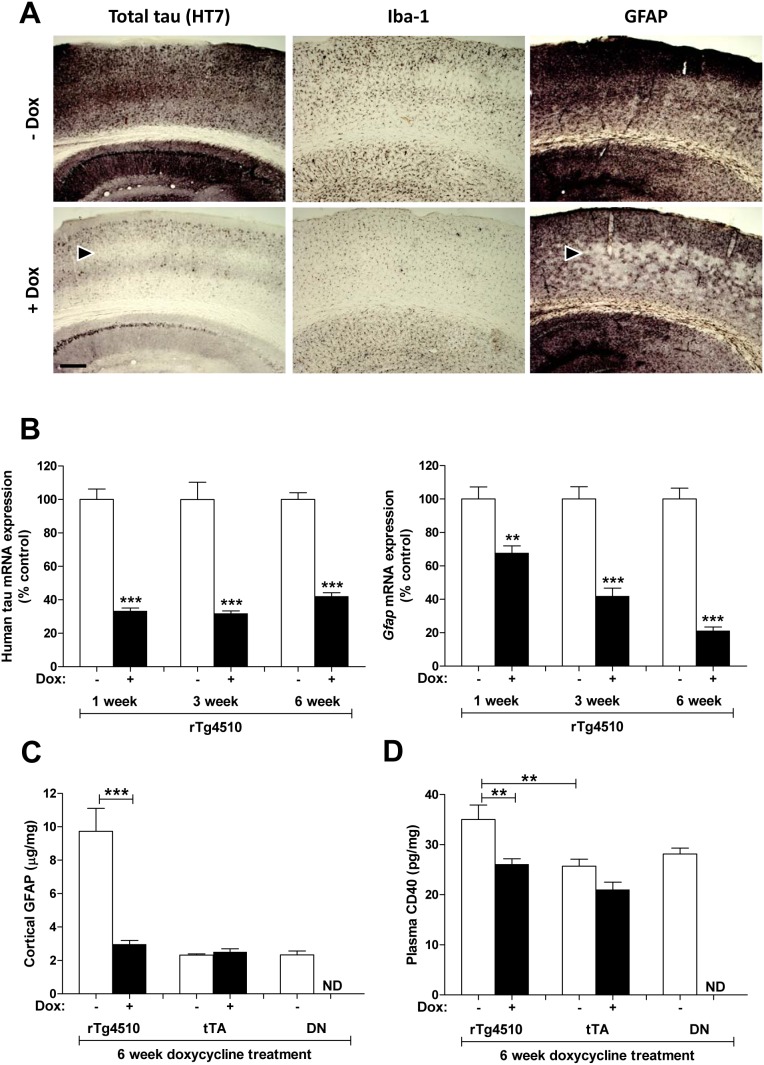
Suppression of tau expression leads to reversal of inflammation. (A) Administration of doxycycline (Dox) to rTg4510 mice resulted in a decrease in total human tau (HT7) in the cortex and hippocampus. The most dramatic reduction was observed in cortical layer IV (arrowhead). Doxycycline administration also resulted in a decrease of Iba-1 and GFAP staining in the cortex. Scale bar, 200 µm. (B) Doxycycline resulted in a decrease in human tau and *Gfap* gene expression measured by qRT-PCR after 1 week of treatment. Further reductions in *Gfap* gene expression were observed with progressively longer treatments prior to 5.3 months of age. (C) Quantitation of GFAP levels by ELISA reveal a 3-fold reduction in rTg4510 mice with 6 weeks of doxycycline treatment, but no change in tTA mice. (D) Plasma levels of the monocyte marker, CD40, were higher in rTg4510 animals than tTA controls, and were suppressed to tTA levels after 6 weeks of doxycycline administration. **p<0.01, ***p<0.001 using the Dunnett multiple comparison test. Error bars indicate SEM.

To further confirm that the inflammatory gene expression changes observed were dependent on tau, 4 month old rTg4510 animals and genotype controls were administered doxycycline in their chow until 5.3 months of age (i.e. 6 week treatment). As expected, levels of human tau (Figure 11A, B) and phospho-tau (data not shown) were greatly reduced with doxycycline treatment. Reduced human tau expression appeared to be most pronounced in a distinct cortical layer (Figure 11A). Surprisingly, astrocyte activation, as measured by GFAP immunohistochemistry (Figure 11A), qRT-PCR (Figure 11B) and ELISA (Figure 11C), as well as microglia activation or monocyte recruitment, as measured by Iba1 immunohistochemistry (Figure 11A), were also reduced with doxycycline treatment. The most robust reduction of GFAP staining appeared to occur in the same cortical layer that showed the most robust reduction in human tau staining (Figure 11A). To get a better idea of whether this reduction was due to prevention of further inflammation or resolution of existing inflammation, rTg4510 mice were treated with doxycycline for 1, 3 or 6 weeks up to 5.3 months of age. Significant reduction in *Gfap* mRNA expression was observed even after 1 week of treatment, with further reduction following longer treatments (Figure 11B), suggesting that neuroinflammation was actually being reversed, not just halted. A similar result was observed for mRNA expression of the myeloid gene, *Cd14*, demonstrating that neuroinflammation caused by multiple cell types was being reversed (data not shown).

### Plasma analytes

Given the robust changes in inflammatory endpoints we observed in rTg4510 brain tissue, we were interested to determine if similar changes also occured in peripheral inflammatory markers. Such analytes could have utility as biomarkers for tracking disease progression or pharmacodynamic responses. 36 analytes were measured in plasma samples using a Rules Based Medicine multiplex assay. Although this assay did not include proteins corresponding to any of the genes that were upregulated in the rTg4510 brain, a number of inflammatory markers were included (see Materials and Methods). Indeed, one inflammatory marker, CD40, was significantly elevated in the plasma of 5.3-month old rTg4510 mice compared to tTA controls (Figure 11D). This elevation was reversed by doxycycline treatment, suggesting that it was dependent on tau expression in the brain (Figure 11D). The CD40 ligand (CD40L) showed similar trends as CD40, though these changes were not statistically significant. No other analytes tested showed doxycycline-reversible differences in rTg4510 mice. While CD40 gene expression was not altered in the brains of rTg4510 mice, CD40 is a marker for monocytes and other inflammatory cells, and may reflect immune activation as a result of signaling from the brain.

### Gene expression profiling of microdissected hippocampal subfields

**Figure 12:**
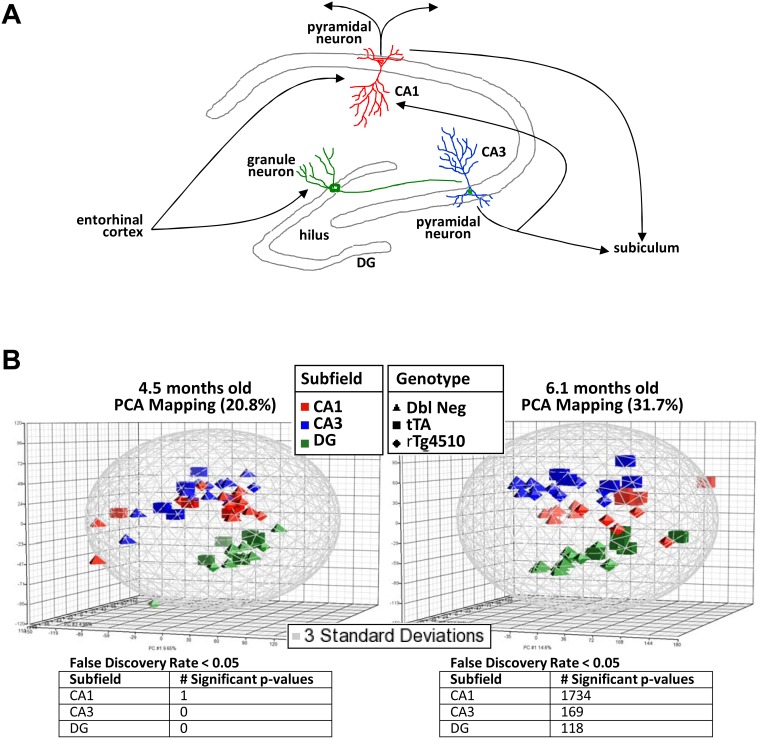
Gene expression profiling of laser microdissected hippocampal subfields. (A) Schematic representation of hippocampal circuitry. Regions isolated by laser capture microdissection are outlined in grey. (B) Principle Component Analysis (PCA) of gene expression profiling data of laser microdissected hippocampal subfields from 4.5 month and 6.1 month old rTg4510 animals and controls. The different subfields, designated by colors, separate from each other along the second principle component (z axis), whereas the genotypes, designated by shapes, separate along the first principle component (x axis), particularly at 6.1 months of age. The tables indicate the number of probe sets that meet the false discovery rate cut-off of 0.05 at the two ages.

**Figure 13:**
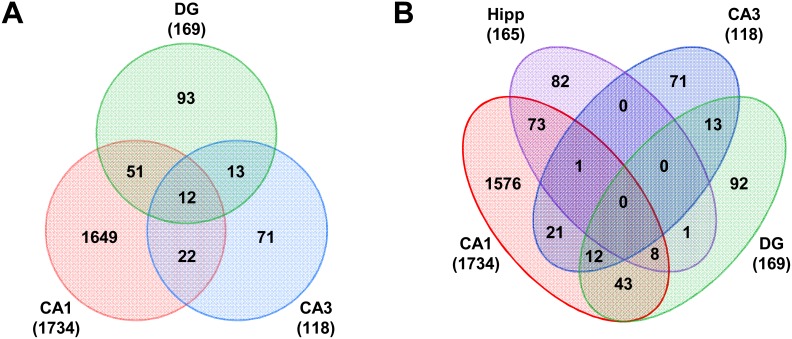
Overview of gene expression changes identified in microdissected hippocampal subfields. (A) Venn diagram of number of differentially expressed genes in the CA1, CA3 and DG microdissected samples. Most of the gene expression changes were observed in the CA1 region, with only modest overlap with the other two regions. (B) Venn diagram of the number of differentially expressed genes in the hippocampal subfields with the whole hippocampus.

**Figure 14:**
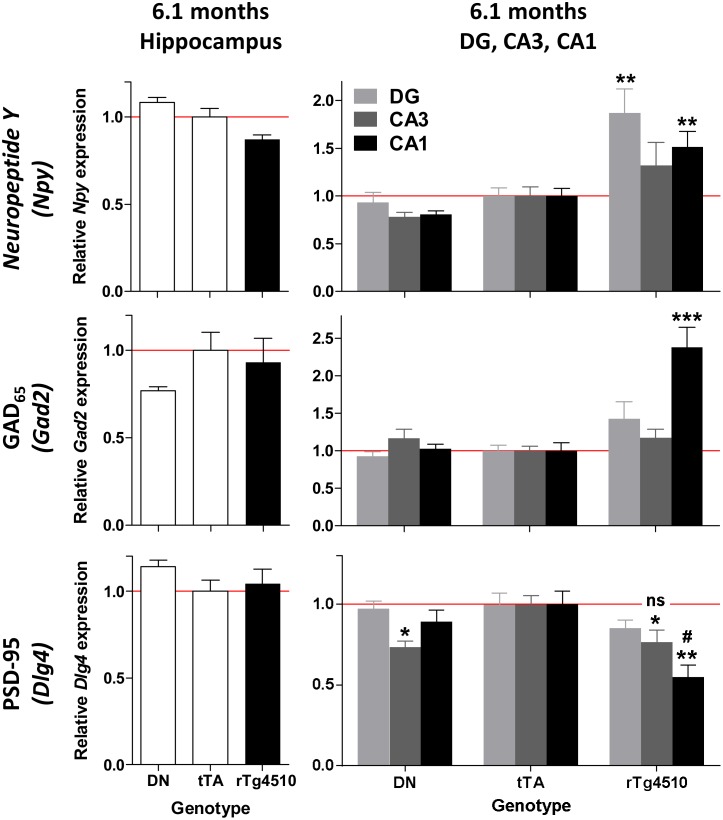
Neuronal alterations in specific hippocampal subfields. Markers for inhibitory neurons, *Npy* and *Gad2*, were elevated in the CA1 pyramidal layer at 6.1 months of age, but no alterations were observed in the hippocampus as a whole. *Npy* levels were also elevated in the dentate gyrus (DG). The postsynaptic marker, *Dlg4*, was decreased in the CA1 pyramidal layer compared to tTA animals. mRNA expression levels are all normalized to tTA. *p<0.05, **p<0.01, ***p<0.001 compared to tTA, ^#^p<0.05, compared to DN, using the Dunnett multiple comparison test. Error bars indicate SEM.

To examine gene expression alterations in the hippocampus at higher resolution, the cellular layers of the major hippocampal subfields–the pyramidal layers of *cornu ammonis* 1 and 3 (CA1 and CA3) and the granular layers of the dentate gyrus (DG)–were isolated by laser microdissection and subjected to transcriptional profiling (Figure 12A). In contrast to the whole hippocampus, very few gene expression alterations were observed at 4.5 months of age in any of the three subfields (Figure 12B). This disparity with the whole hippocampal results may be due to the contributions of regions not included in the laser microdissection, such as the hilus, which contain few neuronal cell bodies but are rich in glial cells. In contrast, dramatic changes were observed at 6.1 months of age (Figure 12B). 86% of these changes (1,734 out of 2,021) were observed in the CA1 pyramidal region (Figure 13A, Tables S4, S5, S6 in File S1), consistent with the observation that neuronal loss and tau pathology occurs predominantly in this subfield. While many of the inflammatory changes observed in the whole hippocampus were also observed in individual subfields (Tables S4, S5, S6 in File S1, Figure S2), certain additional gene expression differences were uncovered (Figure 13B), presumably because the mRNA isolated from the pyramidal and granule cell layers would be enriched for neurons. For example, markers of inhibitory interneurons, such as *Gad2*, which encodes GAD65 (glutamate decarboxylase 2 brain 65 kDa), and the Neuropeptide Y gene, *Npy,* were elevated predominantly in CA1 at 6.1 months, but elevation was not observed in the whole hippocampus (Figure 14). In addition, expression of *Dlg4*, the gene encoding the post-synaptic protein PSD-95, was decreased in the CA1 pyramidal region at 6.1 months of age, but no decrease was detected in the whole hippocampus (Figure 14). These alterations are suggestive of increased inhibitory tone and synaptic loss in the CA1 pyramidal layer, respectively, and would be expected to result in functional behavioral deficits in the rTg4510 mice.

**Figure 15:**
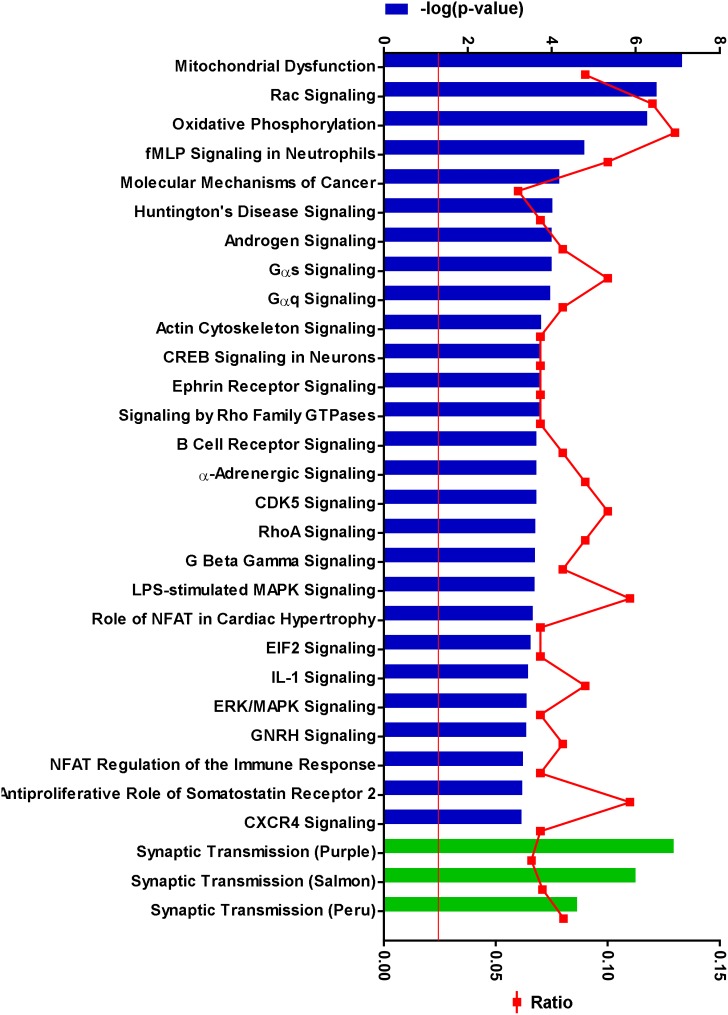
Pathways affected by genes downregulated in the CA1 region. 624 genes downregulated in the CA1 subregion of rTg4510 were subjected subjected to Ingenuity Pathway Analysis. The top axis represents the –log(p-value) of the probability that the gene set represented the canonical pathway by chance, as determined by the Fisher’s Exact Test, with the red line delineating p = 0.05 threshold, and the bottom axis represents the ratio of the number of genes from the query gene set in the canonical pathway to the total number of genes in the pathway. The top canonical pathways significantly overrepresented by these genes, shown by the blue bars, included pathways implicated in neurodegeneration, neuronal signaling and cell migration. The green bars show that these genes also significantly overlap with synaptic transmission modules affected in AD [14]. The query genes in these pathways are shown in Table S8 in File S1.

To further explore the implications of the gene expression changes observed in the hippocampal subfields, genes identified in laser microdissected samples were subjected to pathway analysis. As was the case for the whole hippocampus, genes with altered expression in CA1 overlapped significantly with gene expression modules identified in AD using WGCNA [14], though interestingly the significance was more for synaptic transmission modules and less for the microglial modules compared to the whole hippocampus (Table S7 in File S1, see Figure 6). When upregulated and downregulated genes in CA1 were analyzed separately, the significance of the overlap with the microglial and synaptic transmission modules, respectively, improved drastically (see Figure 6, 15). Downregulated genes overrepresented a number of canonical pathways relevant to neurodegenerative disease, such as mitochondrial dysfunction, as well as pathways important for neuronal signal transduction and cell migration (Figure 15, Table S8 in File S1).

### Behavior

#### Locomotor activity

**Figure 16:**
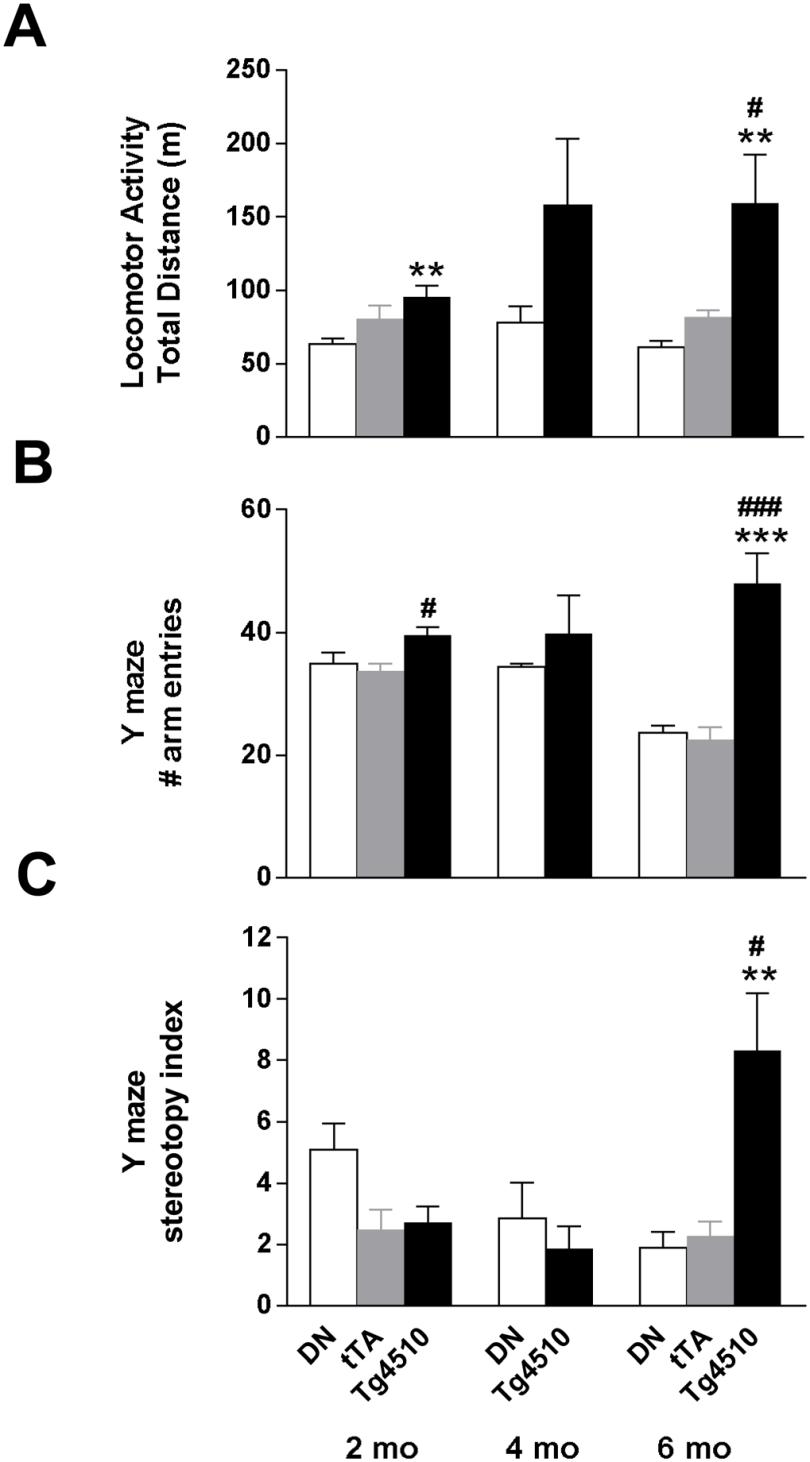
Motoric measures as a function of age in rTg4510 mice. (A) Female rTg4510 mice displayed small but detectable increases in locomotor activity by 2 months of age in a novel locomotor chamber which progressively increased by 6 months of age. (B) In the Y maze, rTg4510 mice displayed a similar increase in locomotion at 2 mo of age and robust increase in locomotion and (C) stereotypy were detected at 6 months of age. n = 7–15/group. *p<0.05, **p<0.01, ***p<0.001 vs DN and #p<0.05, ##p<0.01, ###p<0.001 vs tTA using Tukey’s multiple comparison test. Error bars indicate SEM.

We next wanted to determine whether the gene expression changes observed in the brains of rTg4510 mice correlated with deficits in neuronal funciton that could be detected by behavioral phenotypes. Female rTg4510 mice exhibited a robust and age-dependent increase in locomotor activity (Figure 16A). A genotype effect was detected by 2 months of age (F_(2,33)_ = 5.2, p<0.05), with rTg4510 mice exhibiting an increase in distance travelled compared to DN mice (p<0.01). tTA mice were not different from DN or rTg4510 mice, suggesting an intermediate phenotype. At 4 months of age, rTg4510 mice also appeared to be more active than DN mice although the genotype effect was not statistically significant. By 6 months of age, the genotype differences became more robust (F_(2,42)_ = 6.9, p<0.01), with rTg4510 mice exhibiting a significant increase in distance travelled compared to both tTA (p<0.05) and DN mice (p<0.01).

#### Y maze behavior

Similar to hyperactivity detected in the novel locomotor chamber described above, female rTg4510 mice displayed an age-dependent increase in locomotion in the Y maze (Figure 16B, C). A genotype effect on the number of arm entries was detected (F_(2,39)_ = 3.97, p<0.05, Figure 16B), where rTg4510 mice showed a modest but significant increase in the number of arm entries compared to tTA mice (p<0.05), but not compared to DN mice (Figure 16B). At 4 months of age, there was again a nonsignificant increase in number of arm entries made in rTg4510 mice compared to DN mice. By 6 months of age, rTg4510 mice exhibited a robust increase in the number of arm entries made (F_(2,32)_ = 15.2, p<0.0001) showing significantly increased activity compared to tTA (p<0.001) and DN controls (p<0.001), and stereotyped behavior (F_(2,32)_ = 7.2, p<0.001) compared to tTA (p<0.05) and DN controls (p<0.01) (Figure 16C), neither of which showed stereotopy.

**Figure 17:**
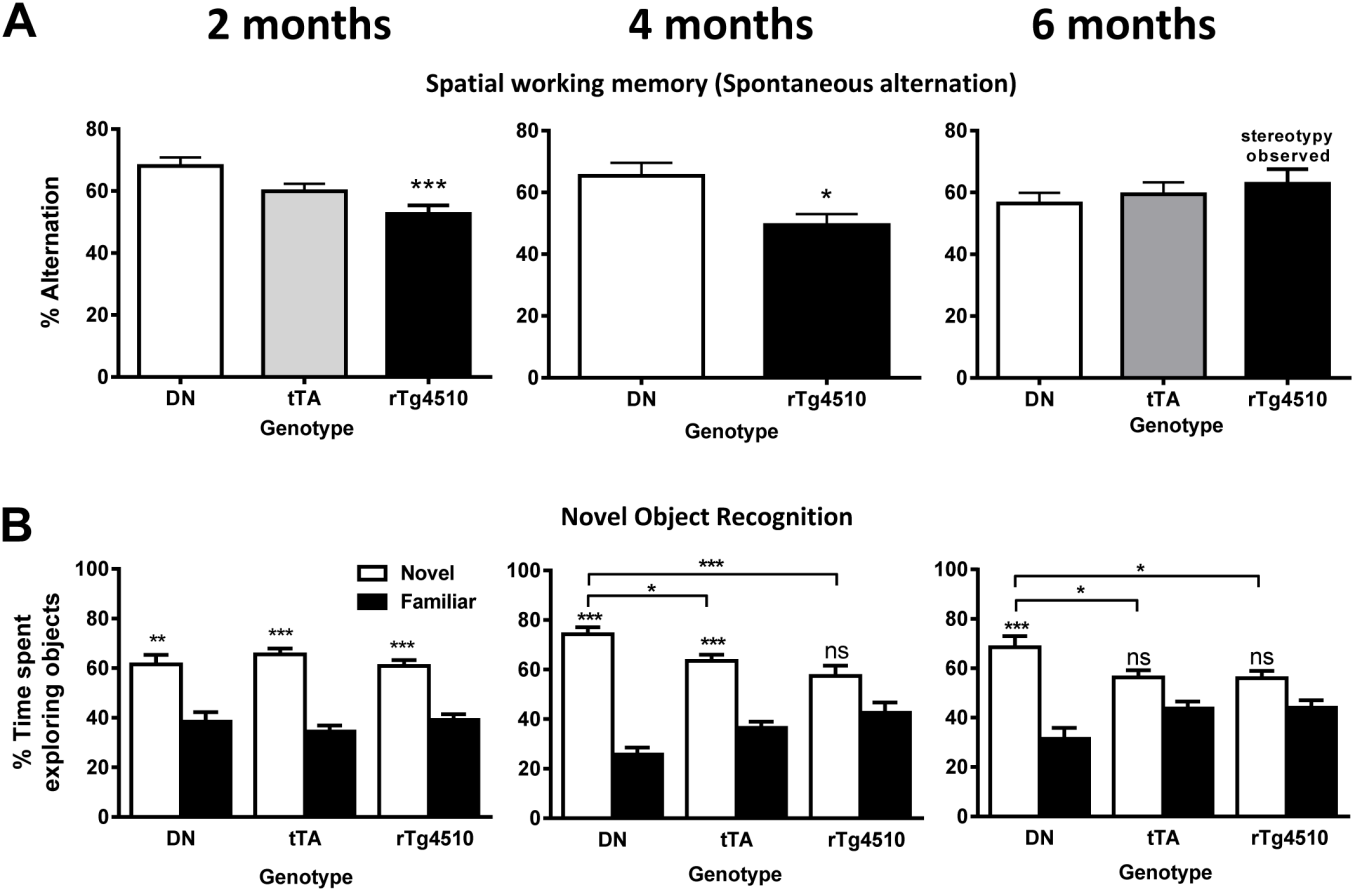
Spatial working memory and object recognition memory in rTg4510 mice. (A) At 2 and 4 months of age, rTg4510 displayed cognitive deficits in spatial working memory compared to DN (1-way ANOVA followed by Tukey’s post-hoc ***p<0.001; student’s t-test *p<0.05). By 6 months of age, spatial working memory was confounded by stereotypic behavior observed in rTg4510 mice. n = 10–12/group. (B) At 2 months of age, all 3 genotypes displayed a preference for the novel object over the familiar object (2-way ANOVA, Bonferroni’s post-hoc **p<0.01, ***p<0.001). By 4 months of age, there was no significant difference in the amount of time rTg4510 mice explored the novel object compared to the familiar object, and the percent of time that rTg4510 explored the novel object was significantly less than the time spent by DN animals (2-way ANOVA, Bonferroni’s post-hoc *p<0.05, ***p<0.001). By 6 months of age, both rTg4510 and tTA animals explored the novel object less than DN animals (2-way ANOVA, Bonferroni’s post-hoc *p<0.05, ***p<0.001). n = 12–15/group. Error bars indicate SEM.

With respect to short term spatial working memory, robust deficits were observed as early as 2 months of age (F_(2,39)_ = 8.7, p<0.001, Figure 17A) with rTg4510 mice alternating significantly less than DN mice (p<0.001). tTA mice were not statistically different from DN or rTg4510. A deficit in spatial working memory was also present at 4 months of age in rTg4510 mice compared to DN mice (t = 2.9, p<0.05); however, at 6 months of age data could not be interpreted due to the confound of stereotyped behavior displayed in this task by the rTg4510 mice.

#### Novel Object Recognition

At 2 months of age, all genotypes showed a significant preference for the novel object over the familiar object (F_(1,42)_ = 55.0, **p<0.01; ***p<.0.001), indicating good short term memory for the object seen previously (Figure 17B). By 4 months of age, there was a significant interaction between genotype and percent time on object (F_(2,31)_ = 7.3; p<0.01). Within group analyses showed that rTg4510 mice had no significant preference for the novel over familiar object whereas DN (**p<0.001) and tTA mice (***p<0.001) retained a strong preference for the novel object. Compared to the DN group, rTg4510 mice showed a robust reduction in percentage of time on the novel object (p<0.001), and tTA mice showed some reduction (p<0.05). At 6 months of age, a significant interaction between genotype and percent time on object was again detected (F_(2,39)_ = 4.1; p<0.05). Within group analyses showed that rTg4510 and tTA mice had no significant preference for the novel over familiar object (Figure 17B), whereas DN mice show a robust preference for the novel object (p<0.001). Compared to DN mice, rTg4510 and tTA mice spent significantly less time on the novel object (p<0.05). The discrimination ratio, a similar index of object recognition memory, again revealed an age-dependent cognitive deficit in Tg4510 mice. No significant differences were detected at 2 months of age, while rTg4510s were significantly impaired compared to DN mice at 4 (p<0.01) and 6 months of age (p<0.05). tTA mice also showed impairment at 4 (p<0.05) and 6 months of age (nsd). Time spent exploring objects were similar for all genotypes during training and testing sessions at all age groups, indicating that cognitive deficits were not related to changes in overall time spent exploring objects during the training or test session.

## Discussion

Elevated levels of phosphorylated tau and neurofibrillary tangles lead to neurotoxicity, cognitive deficits, and behavioral symptoms in the class of neurodegenerative disorders collectively called tauopathies. To better understand the molecular events leading to pathology and neurotoxicity, we took an unbiased approach to characterize gene expression over time in the rTg4510 mouse, and to correlate these changes with functional consequences on behavioral phenotypes.

Strikingly, the largest and most significant gene expression changes overlapped extensively with the immune function networks that were recently identified in AD by connecting genes with related expression signatures and overlaying correlations with clinical severity [12]–[14], [20], [39]. These networks are likely to encompass a number of cell types, including microglia, astrocytes, infiltrating monocytes, neurons, and perhaps lymphocytes and other inflammatory cells. Increased expression of these genes could represent an increase in the number of cells in which they are expressed, an upregulation of expression in existing cells, or a combination of both. Gene expression networks in rTg4510 mice generated by IPA appeared very similar to those identified in AD, with complement pathways representing a major subnetwork centered around a hub formed by the gene, *Tyrobp*. While complement activation is a well-described pathological feature of AD, the role of complement in neurodegenerative disease has received renewed interest as genome-wide association studies (GWAS) have identified alleles of the gene encoding complement component (C3b/C4b) receptor 1, *CR1*, as conferring substantial risk for AD [18], [40]–[44]. Furthermore, *Tyrobp*, which encodes for the protein DAP12, has consistently been identified as the hub of the complement subnetwork of the microglial network [12], [14], [39]. In addition, *Tyrobp* is the coreceptor for *Trem2*, which has also been genetically associated with AD risk [12], [16], [17], [39]. Both genes are amongst the top upregulated genes in rTg4510 in this study and are closest neighbors by hierarchical clustering, suggesting coregulation.

Age-dependent increases in neuroinflammatory genes also coincided with age-dependent decreases in markers of synaptic activity, such as *Arc* and *Homer2,* [45]–[47], and upregulation of markers of inhibitory interneuron activity in the hippocampus. Markers of synaptic activity have been used as surrogates for cognitive function as changes in these markers have been reported to correlate with cognitive deficits in several APP transgenic mouse models [9], [48]. Interestingly, laser dissection of the subfields of the hippocampus from 6.1 month-old rTg4510 mice highlight a robust increase in inflammatory markers specifically in the CA1 region in conjunction with decreased expression of the gene encoding PSD-95 and increased expression of the inhibitory neuronal genes, *Gad2* and *Npy*. The pattern of gene changes observed suggests a possible upregulation of inhibitory activity to compensate for excessive neuronal activation [49]. Dysfunction of inhibitory and excitatory connections which impinge on the CA1 region of the hippocampus may therefore contribute to behavioral disinhibition and cognitive deficits observed at this age.

Interestingly, the locomotor phenotype was both age- and genotype-dependent with robust changes emerging by 6 months of age. Stereotypic behavior also appeared with age in rTg4510 animals. Furthermore, a deficit in short term object recognition memory was also apparent by 4 months of age and was maintained out to 6 months of age. These behavioral deficits build on similar findings in the rTg4510 mice reported by other labs [22], [24], [28], [30], and may represent phenotypes useful for testing functional effects of anti-inflammatory agents. Indeed, reversal of hyperactivity has been shown recently to be a successful measure of functional rescue in rTg4510 mice [28], [50]. The underlying mechanisms of the various behavioral deficits remains unclear, though increased anxiety could be a factor contributing to the increased locomotor activity and stereotypy, and may also interfere with the animal’s ability to perform cognitive tasks. Indeed, anxiety is a significant comorbid symptom observed in Alzeimer’s disease and other neurodegenerative disorders [51]–[53]. The possible role of anxiety in the motor and cognitive phenotypes in rTg4510 mice merits further exploration.

While age-dependent phenotypes could result from either impairment of hippocampal circuits as described here, it is also possible that deficits are related to progressive impairment in neuronal dysfunction in other brain regions. For example, parahippocampal regions have been shown to be sufficient for mediating object recognition in rodents and primates at short delays [54], [55]. In addition, rTg4510 mice show tau expression in the striatum [22] and stereotopies characterized by pronounced repetitive behavior are known to be linked to dysfunction of the motor cortico-striatal loop [56]. Surprisingly, not all behaviors showed an age-dependent deterioration. Deficits in short term spatial working memory, were already robust at 2 months of age and did not appear to show further deterioration over time, although changes in locomotor activity prevented clear assessment of this domain at later ages Since short term spatial working memory is regulated by frontal cortex and hippocampus [35], [36], deficits in this cognitive domain would have been expected to correspond with age-dependent changes in pathology observed. These results raise the possibility that early deficits may stem from an effect of tau overexpression during development. These initial findings demonstrate that a number of functional deficits can be observed in the rTg4510 mice; however, care must be taken in selecting relevant behavioral endpoints to use for future functional studies.

Of note, tTA animals sometimes exhibited an intermediate phenotype between DN and rTg4510 genotypes, suggesting that the tTA transgene could be contributing to some of the deficits in rTg4510 mice. Neurotoxicity and behavioral deficits have previously been reported in the tTA single transgenic animals [57], [58]. Therefore, it is important to use the tTA genotype as a control in all behavioral tests. Indeed, tTA animals showed a number of gene expression changes compared to DN animals (see Figure 1C). This could be due to insertional mutagenesis effects, direct toxicity of the tTA protein product, or interference of the tTA transcriptional activation function with endogenous transcriptional machinery.

While we cannot conclude that inflammation is contributing to the pathology and behavioral deficits, our results suggest that rTg4510 mice could serve as an appropriate preclinical model to test a variety of anti-inflammatory mechanisms for the treatment of tauopathies. Indeed, overexpression of fractalkine, which would be predicted to inhibit microglial activation, has been shown to reverse behavioral deficits in rTg4510 animals [28]. Furthermore, inflammatory markers could serve as invaluable pharmacodynamic endpoints in this model, because the downregulation of tau with doxycycline resulted in reduced inflammation. Therefore, inflammatory markers could be used as surrogate efficacy endpoints to monitor treatments directed at tau, such as tau phosphorylation, in addition to inflammatory treatments. Doxycycline reduced inflammatory markers in as little as one week when administered to animals that already displayed robust inflammation, suggesting the treatment was not just preventing further inflammation, but resolving extant inflammation. The possibility does remain, however, that doxycyline is reducing inflammation directly, since doxycycline has been reported to have anti-inflammatory properties in its own right [59]–[62]. However, the observation that doxycycline appears to preferentially reduce human tau levels in a particular cortical layer and this is the same layer that shows the greatest reduction in GFAP staining suggests that at least some of the reduced inflammation observed with doxycycline treatment was a result of tau reduction.

In summary, we have conducted a characterization of the natural history of tau-dependent changes in gene expression in the rTg4510 mice. The predominant class of gene expression changes were in inflammatory genes, which increased in an age-dependent manner, coinciding with increases in tau phosphorylation, decreases in markers of neuronal activity, and behavioral deficits. These inflammatory genes formed a network highly reminiscent of the immune function gene expression network perturbed in AD. Laser microdissection of hippocampal subfields revealed additional gene expression changes in AD-relevant synaptic transmission pathways. These perturbations may contribute to the observed behavioral deficits. Overall these data suggest that the rTg4510 recapitulates several aspects of human neurodegenerative disease, and could be used as a reasonable preclinical model to test disease-relevant hypotheses and evaluate candidate therapeutic interventions.

## Supporting Information

Figure S1 **CD11b Immunohistochemistry.** Cortical staining for the microglial marker, CD11b, showed a modest increase as a function of age in rTg4510 animals, but remained unchanged in tTA animals. Scale bar, 200 µm, inset scale bar, 50 µm.
(TIF)

Figure S2 **Expression of inflammatory genes in laser microdissected hippocampal subfields.** *C4b*, *Gfap* and *Spp1* mRNA expression levels were higher in 6.1 month old rTg4510 animals in the CA1 hippocampal subfield, as determined by Affymetrix expression profiling. *C4b* and *Gfap* were also significantly elevated in the CA3 region, and *Spp1* in the DG, albeit to a lesser extent. *C4b* was significantly elevated in the DG of tTA mice compared to DN mice, and *Gfap* was elevated in the CA3 and CA1 regions of tTA mice compared to DN mice. mRNA expression levels are all normalized to tTA. *p<0.05, **p<0.01, ***p<0.001 compared to tTA using the Dunnett multiple comparison test. Error bars indicate SEM.
(TIF)

File S1 **This file contains Table S1–Table S8. Table S1. Age-dependent gene expression changes in rTg4510 female hippocampus.** List of the 165 probe sets that show altered age-dependent expression in rTg4510 animals, but not in controls, sorted by fold change between 6.1 month old rTg4510 animals and tTA controls. The presence of each gene in the microglial and synaptic transmission modules identified by Weighted Gene Coexpression Network Analysis (WGCNA) in AD patients [14] is indicated. **Table S2. Inflammatory response pathways altered with age in rTg4510 female hippocampus.** Inflammatory response pathways were the most significantly affected disease and biological functions represented by genes with age-dependent expression changes in the hippocampi of rTg4510 females. The activation z-score, calculated in IPA, predicts the direction of change for the function, with an absolute z-score≥2 considered significant. The number and identity of the molecules in the query set are indicated. **Table S3. Pathways altered with age in rTg4510 female hippocampus.** Inflammatory pathways were the most significantly affected canonical pathways represented by the genes with age-dependent expression changes in the hippocampi of rTg4510 females. Microglial gene expression modules identified in AD [14], were also highly represented in this dataset. The p-values indicate the probability that the gene set represented the canonical pathway by chance, as determined by the Fisher’s Exact Test, while the ratio is the quotient of the number of genes from the query gene set in the canonical pathway to the total number of genes in the pathway. **Table S4. Gene expression changes in the CA1 hippocampal subregion of 6.1 month old rTg4510 females.** List of the 1,734 probe sets that show altered expression in the CA1 region of rTg4510 animals compared to controls at 6.1 months, sorted by fold change between rTg4510 animals and tTA controls. If the probe set was also differentially expressed in other regions analyzed in this study, the fold change between rTg4510 and tTA are shown for that brain region. **Table S5. Gene expression changes in the CA3 hippocampal subregion of 6.1 month old rTg4510 females.** List of the 169 probe sets that show altered expression in the CA3 region of rTg4510 animals compared to controls at 6.1 months, sorted by fold change between rTg4510 animals and tTA controls. If the probe set was also differentially expressed in other regions analyzed in this study, the fold change between rTg4510 and tTA are shown for that brain region. **Table S6. Gene expression changes in the DG hippocampal subregion of 6.1 month old rTg4510 females.** List of the 1,734 probe sets that show altered expression in the dentate gyrus of rTg4510 animals compared to controls at 6.1 months, sorted by fold change between rTg4510 animals and tTA controls. If the probe set was also differentially expressed in other regions analyzed in this study, the fold change between rTg4510 and tTA are shown for that brain region. **Table S7. AD-related expression moduels altered in the CA1 region of 6.1 month old rTg4510 femles.** Microglial and synaptic transmission gene expression modules identified in AD [14] significantly altered in the CA1 region of rTg4510 mice. The p-values indicate the probability that the gene set represented the canonical pathway by chance, as determined by the Fisher’s Exact Test, while the ratio is the quotient of the number of genes from the query gene set in the canonical pathway to the total number of genes in the pathway. The genes in the query set from the rTg4510 data are indicated. **Table S8. Pathways downregulated in the CA1 hippocampal region of 6.1 month old rTg4510 females.** Canonical pathways and AD-related synaptic transmission gene expression modules [14] overrepresented by genes that are downregulated in the CA1 subfield of 6.1 month old rTg4510 females compared to tTA controls. The p-values indicate the probability that the gene set represented the canonical pathway by chance, as determined by the Fisher’s Exact Test, while the ratio is the quotient of the number of genes from the query gene set in the canonical pathway to the total number of genes in the pathway.
(XLSX)
