# Correction: Tau Overexpression Impacts a Neuroinflammation Gene Expression Network Perturbed in Alzheimer's Disease

**DOI:** 10.1371/journal.pone.0121509

**Published:** 2015-03-26

**Authors:** The PLOS ONE Staff

The last sentence of the sixth paragraph of the “Gene expression profiling” subsection of the Results should be displayed as the title of the next subsection, “Suppression of tau expression.”

There is an error in the last sentence of the first paragraph under the heading “Y maze behavior” in the “Behavior” subsection of the Results. The correct sentence is: By 6 months of age, rTg4510 mice exhibited a robust increase in the number of arm entries made (F_(2,32)_ = 15.2, p<0.0001) showing significantly increased activity compared to tTA (p<0.001) and DN controls (p<0.001), and stereotyped behavior (F_(2,32)_ = 7.2, p<0.001) compared to tTA (p<0.05) and DN controls (p<0.01) (Figure 16C), neither of which showed stereotypy.

There is an error in the third sentence of the fifth paragraph of the Discussion. The correct sentence is: In addition, rTg4510 mice show tau expression in the striatum [22] and stereotypies characterized by pronounced repetitive behavior are known to be linked to dysfunction of the motor cortico-striatal loop [56].

There is an error in the legend for Fig. 6. “Mode” should be “most.” Please see the complete, corrected Fig. 6 here.

**Fig 6:**
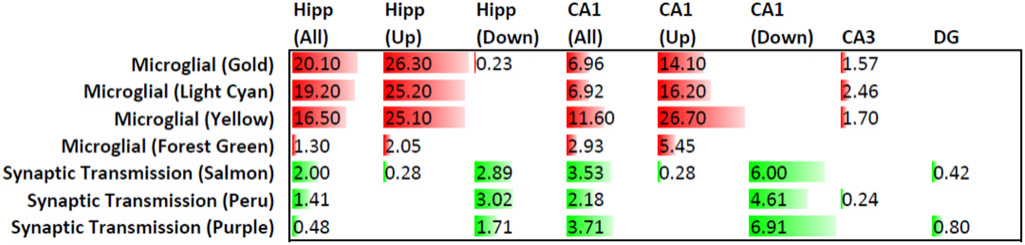
Relationship of gene expression changes in rTg4510 compared to AD brains. Genes with differential expression in rTg4510 brains were compared to gene expression modules affected in AD [14] using the Fisher’s Exact Test. Query samples included all the 139 differentially expressed genes in hippocampus, “Hipp (All)”, the 82 that were upregulated, “Hipp (Up)”, the 57 that were downregulated, “Hipp (Down)”, as well as the genes that were impacted in the CA1, DG, and CA3 microdissected samples. Numbers represent the probability, expressed as -log(p) values, that the query gene sets overlapped with the AD gene expression modules by chance, with -log(p) >1.30 (i.e. p<0.05) serving as the cutoff for significance. When no numbers are indicated, no genes in the query set were present in the modules. The microglial modules (shown in red) were most affected in the Hipp (All), Hipp (Up) and CA1(Up) datasets, whereas the Synpatic Transmission modules (shown in green) were most affected in the Hipp (Down), CA1(All) and CA1(Down) datasets. Gene expression modules identified by weighted gene correlation network analysis (WGCNA) are named by assigning arbitrary colors [11], in this case gold, light cyan, yellow, forest green, salmon, peru and purple.

There is an error in the legend for S1 File. The Table S6 section should state that there were 118 probe sets, instead of 1,734. Please view the corrected S1 File below.

## Supporting Information

S1 File This file contains Table S1–Table S8. **Table S1. Age-dependent gene expression changes in rTg4510 female hippocampus.** List of the 165 probe sets that show altered age-dependent expression in rTg4510 animals, but not in controls, sorted by fold change between 6.1 month old rTg4510 animals and tTA controls. The presence of each gene in the microglial and synaptic transmission modules identified by Weighted Gene Coexpression Network Analysis (WGCNA) in AD patients [14] is indicated. **Table S2. Inflammatory response pathways altered with age in rTg4510 female hippocampus**. Inflammatory response pathways were the most significantly affected disease and biological functions represented by genes with age-dependent expression changes in the hippocampi of rTg4510 females. The activation z-score, calculated in IPA, predicts the direction of change for the function, with an absolute z-score≥2 considered significant. The number and identity of the molecules in the query set are indicated. **Table S3. Pathways altered with age in rTg4510 female hippocampus**. Inflammatory pathways were the most significantly affected canonical pathways represented by the genes with age-dependent expression changes in the hippocampi of rTg4510 females. Microglial gene expression modules identified in AD [14], were also highly represented in this dataset. The p-values indicate the probability that the gene set represented the canonical pathway by chance, as determined by the Fisher’s Exact Test, while the ratio is the quotient of the number of genes from the query gene set in the canonical pathway to the total number of genes in the pathway. **Table S4. Gene expression changes in the CA1 hippocampal subregion of 6.1 month old rTg4510 females**. List of the 1,734 probe sets that show altered expression in the CA1 region of rTg4510 animals compared to controls at 6.1 months, sorted by fold change between rTg4510 animals and tTA controls. If the probe set was also differentially expressed in other regions analyzed in this study, the fold change between rTg4510 and tTA are shown for that brain region. **Table S5. Gene expression changes in the CA3 hippocampal subregion of 6.1 month old rTg4510 females**. List of the 169 probe sets that show altered expression in the CA3 region of rTg4510 animals compared to controls at 6.1 months, sorted by fold change between rTg4510 animals and tTA controls. If the probe set was also differentially expressed in other regions analyzed in this study, the fold change between rTg4510 and tTA are shown for that brain region. **Table S6. Gene expression changes in the DG hippocampal subregion of 6.1 month old rTg4510 females**. List of the 118 probe sets that show altered expression in the dentate gyrus of rTg4510 animals compared to controls at 6.1 months, sorted by fold change between rTg4510 animals and tTA controls. If the probe set was also differentially expressed in other regions analyzed in this study, the fold change between rTg4510 and tTA are shown for that brain region. **Table S7. AD-related expression moduels altered in the CA1 region of 6.1 month old rTg4510 femles**. Microglial and synaptic transmission gene expression modules identified in AD [14]significantly altered in the CA1 region of rTg4510 mice. The p-values indicate the probability that the gene set represented the canonical pathway by chance, as determined by the Fisher’s Exact Test, while the ratio is the quotient of the number of genes from the query gene set in the canonical pathway to the total number of genes in the pathway. The genes in the query set from the rTg4510 data are indicated. **Table S8. Pathways downregulated in the CA1 hippocampal region of 6.1 month old rTg4510 females**. Canonical pathways and AD-related synaptic transmission gene expression modules [14] overrepresented by genes that are downregulated in the CA1 subfield of 6.1 month old rTg4510 females compared to tTA controls. The p-values indicate the probability that the gene set represented the canonical pathway by chance, as determined by the Fisher’s Exact Test, while the ratio is the quotient of the number of genes from the query gene set in the canonical pathway to the total number of genes in the pathway.
(XLSX)
