# Supplementary figures and images for: Tau Overexpression Impacts a Neuroinflammation Gene Expression Network Perturbed in Alzheimer’s Disease
Source: PLoS One. 2014 Aug 25;9(8):e106050. doi: 10.1371/journal.pone.0106050 (PMC4143352; doi:10.1371/journal.pone.0106050)

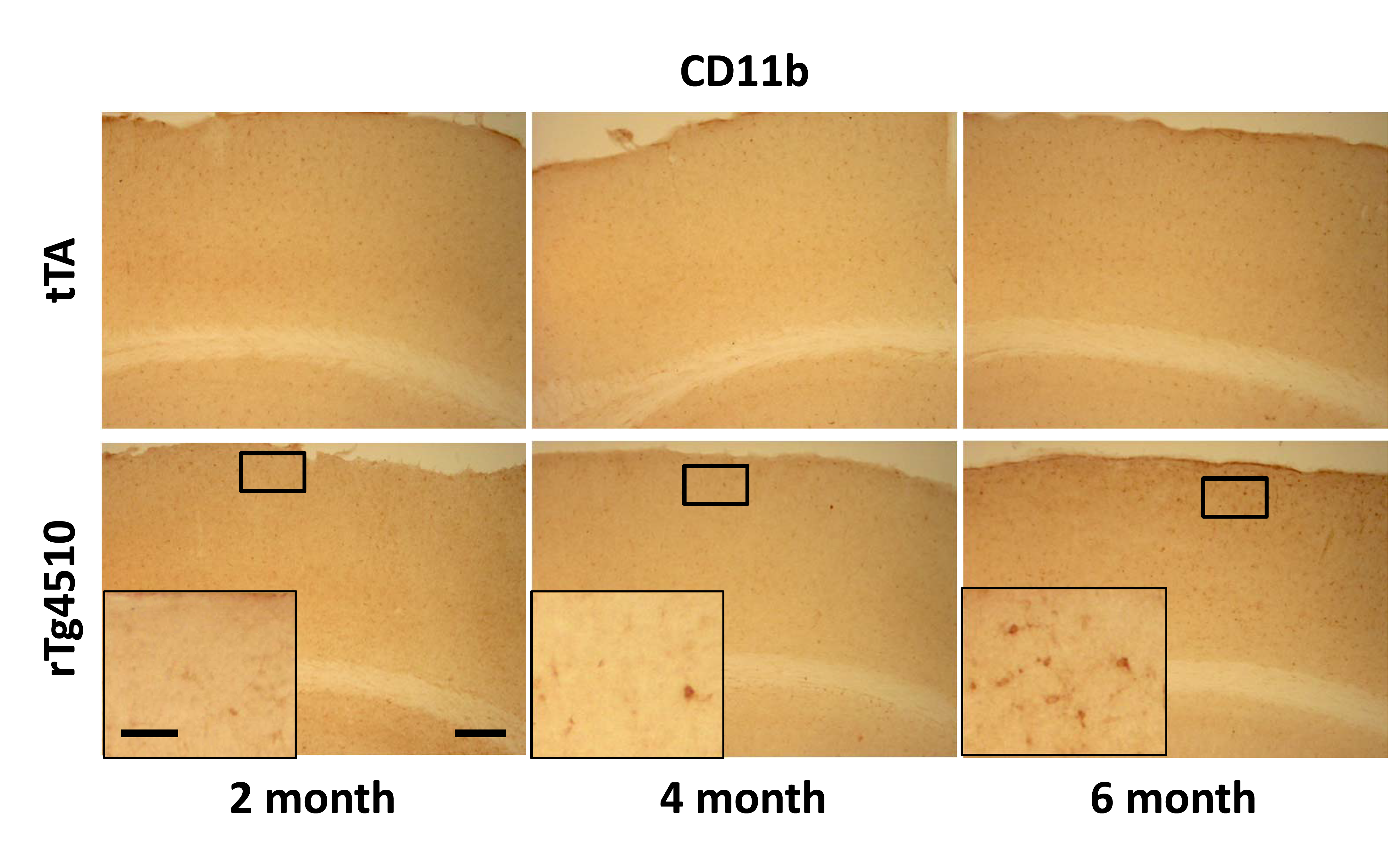

Supplement: Figure S1 — CD11b Immunohistochemistry. Cortical staining for the microglial marker, CD11b, showed a modest increase as a function of age in rTg4510 animals, but remained unchanged in tTA animals. Scale bar, 200 µm, inset scale bar, 50 µm. (TIF) [file pone.0106050.s001.tif]

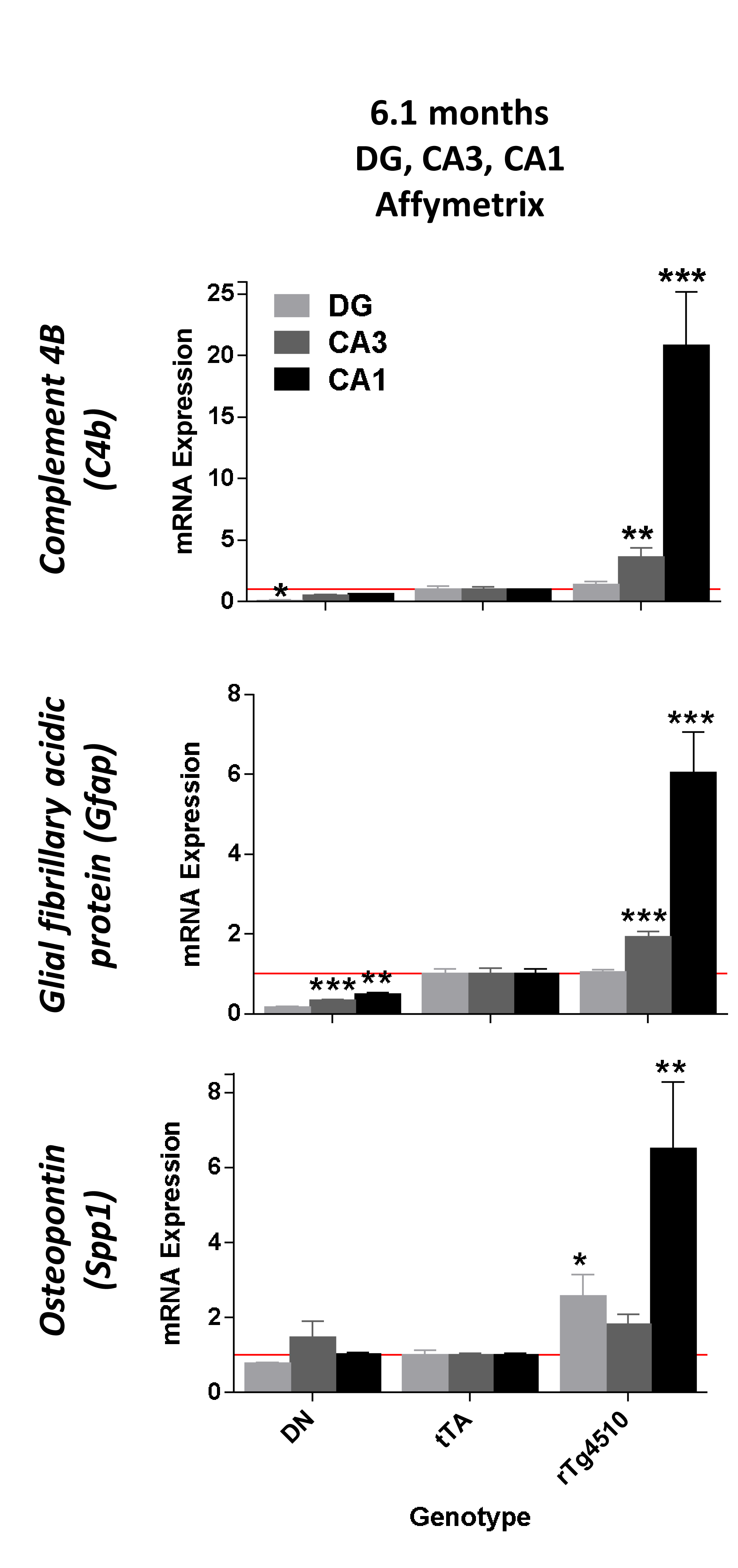

Supplement: Figure S2 — Expression of inflammatory genes in laser microdissected hippocampal subfields. C4b, Gfap and Spp1 mRNA expression levels were higher in 6.1 month old rTg4510 animals in the CA1 hippocampal subfield, as determined by Affymetrix expression profiling. C4b and Gfap were also significantly elevated in the CA3 region, and Spp1 in the DG, albeit to a lesser extent. C4b was significantly elevated in the DG of tTA mice compared to DN mice, and Gfap was elevated in the CA3 and CA1 regions of tTA mice compared to DN mice. mRNA expression levels are all normalized to tTA. *p<0.05, **p<0.01, ***p<0.001 compared to tTA using the Dunnett multiple comparison test. Error bars indicate SEM. (TIF) [file pone.0106050.s002.tif]
